# Comparing causal techniques for rainfall variability analysis using causality algorithms in Iran

**DOI:** 10.1016/j.heliyon.2018.e00774

**Published:** 2018-09-11

**Authors:** Majid Javari

**Affiliations:** College of Social Science, Payame Noor University, PO Box 19395-3697, Tehran, Iran

**Keywords:** Atmospheric science, Environmental science

## Abstract

Causal analysis (CA) is a strong quantitative approach whose mechanisms have climatic predictions. In this study, we studied the patterns of causality (PC) on the effect of rainfall (ER) using climatic series collected from 170 stations for the period 1975–2014 in Iran. Next, we predicted the causal relationships of climatic variables using causal models, including first-generation techniques (FGT), second-generation techniques (SGT), third-generation techniques (TGT), and causal hybrid techniques (CHT). Then, we estimated the causal models using partial squares algorithms (PSA), mechanical equations modeling algorithms (MEMA) such as exploratory and confirmatory methods, and spatial variability methods such as geostatistics and spatial statistical methods. Finally, we evaluated the quality of the methods using the goodness of fit indices, including absolute fit indices (AFI), comparative fit indices (CFI), and parsimonious fit indices (PFI). The results showed that CHT algorithm more suitably predicted the climatic spatiotemporal effect variability (SEV) by extracting direct, indirect, and total effects of climatic variables. Based on the CHT algorithm, the highest and lowest effect values were observed in total effects of winter rainfall (0.98) and summer rainfall variables (0.1), respectively. The SEV ranged from 0.8 to 0.98 for the winter rainfall total effects of CHT in Iran. Using CHT, most of the predicted SEV, particularly the rainfall series, displayed SEV varying from 80% to 98% of the winter rainfall total effects to the annual rainfall in Iran. Similarly, based on the CHT, the highest and lowest SEV values were in western, eastern, and southern regions and in central regions, respectively. In addition, the SEV varied within the range of 0.6–0.74 (varying from 60% to 74% for the autumn rainfall total effects of the annual rainfall in Iran) for the autumn rainfall total effects in Iran. Finally, the SEV of this type of analytical pattern as well as designated subject of CA applications in the atmospheric science and environmental science are discussed.

## 1 Introduction

Climatic effect variability (CEV) can represent itself as a direct or indirect technique for pattern scheming of causality made based on the climatic factors and variables (Javari, 2017b). Reproduction of CEV is the important process in climatic variables effect evaluated with the suggested CPSRM, PLS and GIS algorithms in climate (Zheng et al., 2017). In the climatic CPSRM process, rainfall variability is the initial cause of the analysis of climatic effect variability (CEV) at time phases (Javari, 2017f; Läderach et al., 2017). The climate effect differs in various temporal and spatial models. All the climatic models predict that the relationships between climatic factors and variables in different areas are studied to be simulated. Therefore, various models to predict effect variability of climatic factors and variables are used (Cui et al., 2017; Javari, 2017a; Läderach et al., 2017; Li et al., 2017). In recent years, various CPSRM have been applied in analyzing effect of climatic factors and variables on spatiotemporal levels checking numerous effects in the climate system (Javari, 2017d; Azari et al., 2017; Untenecker et al., 2017; Ciervo et al., 2017; Wang et al., 2017a). However, there are studying facts displaying that climatic variables and suitable factors were not studied (Wang et al., 2017a,b; Hermwille et al., 2017; Frankel et al., 2009; Howarth et al., 2017). In addition, climatologists considered their causal and non-causal effect short time in the past (Wang et al., 2017a), and the application of causal and non-causal effect methods to predict the climatic temporal-spatial variability such as rainfall, temperature and humidity in various regions (Javari, 2017b; Mostafaeipour et al., 2017; Li et al., 2017). The number of causal and non-causal effect models applications in climatic CPSRM changes, and the progress of causal models is well used by various climatic disciplines to some exceptional studies (Hewitt et al., 2017) such as that of Hewitt et al. (2017). However, the subject of causal models such as CPSRM, PLS and GIS has considered only partial application in the climatic CPSRM thus far, thereby diminishing to apply causality models to its detailed ability in its effect to predict the climatic effect variability (CEV) (Hewitt et al., 2017; Bühlmann et al., 2014; Javari, 2017b). Climatic effect variability (CEV) is structured as a multi-effect level according to CPSRM, PLS, and GIS models on climate, and FGT has been formulated a mono-sided analysis pattern on analysis of climatic changes (Tenenhaus et al., 2005). FGT models include the measurement scheme (MS), controlling climate, and effect patterns (EP) (Drton and Maathuis, 2017). Mechanical-causal models (SCM) manages climate and effect patterns (EP). In addition, climatic effect variability (CEV) is structured as a multi-causal level according to CPSRM, PLS and GIS models on climate, and SGT has originated from a monophonic-sided examination pattern on studying climatic changes. SGT models include the reflective technique (RM) and formative technique (FT) controlling climatic latent variables (CLV), and the effect scheme is produced by its own climatic manifest variables (CMS) (Cui et al., 2017). SGT measurement and mechanical-causal models (SCM) manage climate, and effect variability spatially and temporally. The difference between the RM and the FT is the one in the formative technique, and it is considered that the CLV is produced by its particular CMS, while in the reflective technique, it is measured that CMS is formed by its specific CLV (Temme et al., 2006; Henseler and Fassott, 2010). In this study, by referring to “mechanical-causal models of effect”, covariance-based analysis or causal processes and mechanical relations (CPSRM) is a generalized exploratory and confirmatory causal technique (Lomax and Schumacker, 2012), as it is mainly suitable to analyze climatic effect variability (CEV) and systematic relationships of variables in climate (Javari, 2017d). Patterns of partial least squares (PLS) in climatic CPSRM are developed, and the applications of PLS are well applied by some climate disciplines (Javari, 2017b). On the other hand, the subject of PLS statistical applications has been established only to analyze RM and FT applications (Javari et al., 2016; Tenenhaus et al., 2005). Patterns of partial least squares mechanical equation modeling (PLS-CPSRM) in climatic CPSRM are combined, and applications of PLS-CPSRM are well used by some climate subjects. Patterns of PLS-CPSRM in climatic CPSRM are combined to predict direct effects as well as indirect effects and total effects, being well employed by path diagrams and path coefficients in the climate analysis (Javari, 2017b). In analyzing PLS-CPSRM models, factors are the latent variables separated as linear patterns of the observed variables. In one climatic PLS-CPSRM modeling, PLS algorithms (i.e., inner models as phase among path coefficients, total effect coefficients, and indirect effect coefficients, outer models as a sequence of path coefficients and path loadings and constructs as R-square coefficients) consider some essential components. In one climatic CPSRM modeling, CPSRM algorithms (i.e., measurement models as phase among path coefficients, total effect coefficients, and indirect effect coefficients, mechanical models as a sequence of path coefficients and R-square coefficients) consider some essential components. The third scenario has a special method to analyze effect and causality. This method determined a GIS-based spatial modeling of climatic variables using geostatistical techniques as GIS algorithms. The GIS algorithms were also used in the GIS tools in Iran to predict the climatic effect variability (CEV) based on several synthetic maps of the effect spatial variability of climatic variables such as that of Cai et al. (2017). Finally, based on the methods and models in the hybrid algorithm to analyze effect and causality multi-spectral analysis as Comprehensive algorithm, the article objective to reveal how to predict a climatic effect variability (CEV) to simulate the climatic effect spatiotemporal variability aiming at optimizing climatic classification. The purposes of this study are to (1) examine the PLS algorithms properties of climatic variables in Iran, (2) analyze the CPSRM algorithms properties of climatic variables in Iran, (3) explore the GIS algorithms properties of climatic variables in Iran, (4) investigate the hybrid algorithm properties of climatic variables in Iran, and (5) review the results concluding the paper.

## 2 Materials & methods

### 2.1 Study area and data analyses

Iran is located in the southwest of Asia with a 25° 3′–39° 47′ N latitude and 44° 5′–63° 18′E longitude, and it is a mainly diverse landforms realm where two major landforms, the mountainous (highlands) realm and the lowlands (plains) realm (has an elevation range from −26 to 5671 m) (Javari, 2016b), separate different climatic zones (relatively wet climate to dry climate) (Alizadeh-Choobari and Najafi, 2017). Climatic data series during the period from 1975 to 2014 collected from the Meteorological Organization of Iran (http://www.irimo.ir) were employed to predict the climatic effect variability (CEV). In this study, there are 170 climatology and synoptic stations, and data quality control was performed using various methods such as normality, linearity, homogeneity, outliers, and lacking or missing data. As all the series were complete, no reproduced method was required. For each station, data series statistical properties were studied to analyze the CEV properties such as normality, linearity, homogeneity, and outliers. First, various generalized first-generation statistical parameters of data series were estimated for each station for the organized period. Various climatic variables in different stations are used to analyze the CEV in Iran. In this study, the selected data series include climatic variables and factors (annual temperature average, minimum temperature average, maximum temperature average, dew point temperature, daily temperature, annual precipitation, daily precipitation average, monthly precipitation, seasonal precipitation, relative humidity average, maximum relative humidity, minimum relative humidity, saturation ratio, wind speed, cloudiness, vapor pressure, saturation vapor pressure, average pressure, maximum pressure, minimum pressure, and elevation).

### 2.2 Methods

#### 2.2.1 An analysis for understanding causality procedures

First-generation techniques (FGT)falling within the group of statistical methods (descriptive and inferential) theoretically could be used for a class of objectives, but here, the important object is suitable recognition of the performance of latent climatic variables using regression, factor, cluster, correlation, and causal processes and mechanical relations (CPSRM) methods (Lomax and Schumacker, 2012). From a technical viewpoint, these hidden climatic variables are independent variables, like the response (answer) variables, that were directed at the origin of cross-products relating to the response variables (Sorooshian, 2017). While hidden climatic variables may be considered CPSRM-based modeling variables, these CPSRM modeling variables analyze their direct, indirect and total causal effects in the climate system. From a climatic viewpoint, only the observed variables would be considered the climatic effect variability (CEV). CPSRM is a statistical method to assess effects between climatic observed (indicator or manifest) and hidden variables (unobserved variables or factors) (Driver et al., 2017). A FGT define a component-based approach using a type of principal components analysis to construct hidden variables to series analyzed under a specific climatic effect variability (CEV). Furthermore, for climatologists, climatic hidden variables are in contrast to the established CPSRM using a type of ordinary factor analysis to produce climatic hidden variables. However, this background has classified two types of variables: the hidden climatic variables obtained in conceptual models, and the observed variables located in the data series. The CPSRM methods obviously combine a specific climatic type of variable (Lomax and Schumacker, 2012). In this context, the climatic components in the climatic-constructed CPSRM, and the combinations in multiple-climatic causal processes and mechanical relations are collected and analyzed as “multiple-climatic variables” (Javari, 2017b), and well-defined as climatic effect variables (CEVs) used for climatic effect variability (CEV). Climatic-constructed CPSRM and multiple-climatic CPSRM use climatic variables, factors or multiple-climatic variables to predict the effect and causal patterns. These patterns reflect different assumptions (Westland, 2015) concerning the relationship between climatic observed and hidden variables in a data series. Hidden variables reflected in CPSRM can represent a wide variety of climatic relationships in a data series. Similarly, the climatic observed-hidden spatiotemporal distinction also provides a way to take the explanation of effect score reliability. Therefore, in addition to the essential statistical differences between the one-track-climatic-based and complex-climatic based methods for climatic effect variability (CEV), there is an important path modeling similarity (Javari, 2017d; Lomax and Schumacker, 2012). In addition, many basic properties of CPSRM patterns were used in this CPSRM to follow the two main aims: the investigation to recognize patterns of correlations in a climatic data series, and to analyze the data variance as much as possible with the pattern used by the climatologist to understand the causal processes and mechanical relations (CPSRM) (Drton and Maathuis, 2017). The between-group relationships considered within a CPSRM construction (Siied Abaszadeh et al., 2012) are adjusted using forecasted influence to explain a CPSRM model based on experimental conditions to which patterns are spatiotemporally assigned (Nainggolan et al., 2018). In the CPSRM, hidden variables analyzed in CPSRM are usually adopted to be continuous (Westland, 2015). There are various procedures employed in analyzing effect models in the climatic categorical hidden variables. The levels of a multi-hidden variable are named “climatic causal patterns”, and they are a combination of climatic sub-classes suggested from the data series (Hewitt et al., 2017). In other words, this analysis pattern detects quality and type of hidden climatic causal patterns. Analysis of hidden climatic patterns is causal analysis, but for composite observed and latent climatic variables (Bühlmann et al., 2014). A different type of hidden climatic causal pattern representing the change from one of the two different states such as from an interactive relation or common effect to extractive or individual effect of a capacity, is a hidden transition climatic causal pattern (Drton and Maathuis, 2017). Analysis of CPSRM models includes two types of effect analysis for the observed composite and hidden climatic variables. Indirect effects analysis is statistically estimated as the result of direct effects (as path coefficients), either standardized or unstandardized (Javari, 2017g; Markus, 2012). Total effects are the sum of all direct and indirect effects of one variable on another. Total standardized effects are also explained as path coefficients, and unstandardized estimates of total effects are determined similarly, but with unstandardized coefficients (Drton and Maathuis, 2017; Kline, 2015). In the application of first-generation techniques, multivariate normality, the univariate outlier, linearity, homoscedasticity, missing observations, multicollinearity, reliability and validity properties CHT be considered to analyze the specific climatic effect variability (CEV) (Javari, 2017d). In this study, the climatic components in the CPSRM-constructed model and the combinations in the climatic multiple-variables model are analyzed as confirmatory-based analysis (CBA) and exploratory-based analysis (EBA). In CPSRM, they (Javari, 2017b) are considered as climatic effect variables (CEVs) based on the measurement models (the relationships between observed variables or indicators) and hidden variables or climatic factors as mechanical models (the relationships between hidden variables) for climatic effect variability (CEV). In addition, in this study, the climatic components in the climatic-constructed CPSRM and the method in the multiple-climatic CPSRM are used as maximum likelihood-based analysis. In the climatology, climatologists use endogenous and exogenous variables. Exogenous hidden variables are synonymous with independent variables; they “cause” variations in the values of other latent variables in the model. They are reflected to be influenced by other factors external to the model. Endogenous hidden variables are synonymous with dependent variables and therefore are influenced by the exogenous variables in the model, either directly or indirectly (Sageghpour and Moradi, 2009). Measurement and mechanical models are the term used to describe a situation in CPSRM (Javari, 2017b). Partial least squares (PLS) analysis is used as an SGT to predict the climatic effect variability (CEV) with independent and response variables (Temme et al., 2006). PLS is a composite-based CPSRM, and a component-based CPSRM (Henseler and Fassott, 2010; Keller et al., 2014), in comparison to CPSRM, using a set of independent variables to multiple response variables to predict the climatic effect variability (CEV). PLS is two types of mechanical modeling being well-defined as climatic effect variables (CEVs) dealing specifically with reflective models—that is, in the path, diagram arrows go from the hidden variable to the measured indicator variables or climatic factors—as formative components that is, the arrows go from the observed measures to the hidden variables in the climatic effect variability (CEV). Partial least squares regression is an extension of the multiple linear regression model. The standard algorithm to compute PLS components (i.e., factors) is considered as nonlinear iterative partial least squares (NIPALS). Many variants of the NIPALS algorithm exist, which normalize or do not normalize certain vectors. A different assessment method for partial least squares regression factors is the SIMPLS algorithm. To study the climatic effect variability (CEV) in Iran, we used two broad types of measurement specification: reflective and formative measurement models. The reflective measurement model has an extended method in the climatology, and is directly based on standard test hypothesis whose amounts represent effects of a causal hypothesis. As a result, causality is from the paradigm to its amounts. In comparing, formative measurement models (confirmatory and exploratory) are based on the method presented in the climatology that the indicators produce the construct (Temme et al., 2006). To analyze the climatic effect variability (CEV) in Iran, we used combined types of patterns: reflective and formative measurement models (as a hybrid model). The PLS-CPSRM algorithm (hybrid model) assesses all unknown patterns in the PLS path model in the climatic effect variability (CEV) in Iran. After estimating the pattern scores by the algorithm, the scores are used to estimate each PLS model in the path model (Henseler and Fassott, 2010). As a result, we obtain the estimates for all relationships in the measurement and the mechanical models (Hair, 2017). All PLS models are estimated by the PLS- CPSRM algorithms, including two phases. In the first phase, the construct (hypothesis) scores are calculated. Next, in the second phase, final calculators of the outer CHT and loadings (path coefficients and the determination coefficient values) are estimated (Drton and Maathuis, 2017; Hair, 2017). In using PLS- CPSRM, it is valuable to identify that the fit has different values in the frameworks of CPSRM and PLS- CPSRM (Tenenhaus et al., 2005). Fit indicators for PLS-CPSRM are obtained from the difference between the observed and the model-suggested covariance matrix, hidden variables values and values calculated by the suggested model (Markus, 2012; Javari, 2017b). With the considerable change in the scientific technology in climate, new technologies such as GIS has a considerable impact on climatic services and climatic predictions. This article presents a broad GIS-based technology suitable for climatic effect variability (CEV) analysis with different types of applications. The basic functions of the GIS-based technology include geostatistics, spatial statistics, spatial analysis and layer production of maps (Xu and Zhang, 2013). The analysis and forecast of climatic effect variability (CEV) are presented based on the maps of FGT, GSHT, and TGT types. The monitoring CEV patterns control CPSRM models, PLS models, PLS-CPSRM models, and GIS-based PLS- CPSRM models types using ArcGIS. Basic GIS-based PLS-CPSRM models, extracting the maps of climatic effect variability (CEV), were then predicted in point data form using the geostatistical methods using ArcGIS_10.5_ software (Zheng et al., 2017). We estimated climatic effect spatial variability by ArcGIS_10.5_ using Geostatistical Analyst. Since we were required to provide patterns of the climatic effect spatial variability (CEPV) in Iran, we needed to predict the effect patterns of the climatic variables (Javari, 2017c). Geostatistical method is a branch of statistics focusing on spatial or spatiotemporal climatic series. Finally, we predicted the spatial variability of climatic series effect (Armstrong and Champigny, 1989). Geostatistical techniques depend on statistical patterns and methods based on the random function concept to simulate the uncertainty combined with spatial prediction and estimation (Honarkhah and Caers, 2010). Use of GIS-based PLS-CPSRM techniques such as ordinary kriging (OK) and kriging interpolation techniques are some geostatistical analytical tools in ArcGIS_10.5_ essential for climatic effect spatial variability (CEPV). To understand climatic effect spatial variability (CEPV), perfect measurable methods such as GIS-based modeling (Javari, 2017d) and climatic data are needed (Suryabhagavan, 2017). CEPV over the study area was characterized using a set of techniques constructed by a GIS-based PLS-CPSRM project. The GIS-based PLS-CPSRM project focuses on effect patterns spatially and temporally, such as the elevation impact on rainfall (Javari, 2017b). The climatic data series nature assessment was controlled by GIS-based modeling indicators (Westland, 2015). GIS-based PLS-CPSRM project was organized to estimate 20 (absolute fit indices, comparative fit indices, and parsimonious fit indices) indicators to estimate the spatial variability in climatic effect patterns (CEP) over the study period (Javari, 2017g; Schumacker and Lomax, 2004).

#### 2.2.2 Data quality assessment and FGT applications

Climatic data series (annual average temperature, average minimum temperature, average maximum temperature, dew point temperature, daily temperature, annual total precipitation, daily average precipitation, monthly precipitation, seasonal precipitation (winter, spring, summer, and autumn), average relative humidity, maximum relative humidity, minimum relative humidity, saturation ratio, wind speed, cloudiness, vapor pressure, saturation vapor pressure, average pressure, maximum pressure, minimum pressure, and elevation) from 170 stations belonging to the Meteorological Organization (MO) of Iran for GIS-based PLS-CPSRM project analysis (GPMA) were used in Iran. All of the 170 climatology and synoptic stations were collected for analysis based on the long-term data (40 years) and quality with missing data for less than 5 percent. Various climatic variables at various stations were used to analyze the CEV in Iran. In this study, the selected data series included climatic variables and factors. Statistical properties of the data series were analyzed using descriptive statistics through descriptive statistics such as coefficient of variations (CV) to predict the statistical indications to classify extreme changes in the data statistical distribution.

#### 2.2.3 Causal processes-based modeling

Causal processes-based modeling, as a causal processes and mechanical relations model (CPSRM), was initially established by Wold (1982) for casual analysis (Nitzl, 2016). CPSRM is a statistical method to estimate relationships between climatic variables (observed and hidden). To provide the CPSRM-based modeling (Mclntosh and Gonzalez-Lima, 1994; Sorooshian, 2017), we represent climatic variables and components in climatic constructed CPSRM in Iran. The climatic constructed CPSRM divides the measurement model into climatic series and mechanical modeling based on hidden variables (Westland, 2015). The models are constructed, and the components of causal variability for climate classification in Iran are represented. Each path model is depicted by the direct effect of manifest variables to hidden variables and indirect effect of manifest variables to manifest and hidden variables, and total effects for manifest variables to hidden variables and manifest variables. Causal models in CPSRM-based modeling are essentially integrated as climatic variables and components constructed for the causal processes-based project of temporally daily, monthly, seasonal and annual data employed to predict the climatic effect variability (CEV) in Iran. The aim of the CPSRM-based modeling is to predict the effect patterns (EP) of employing three patterns in Iran. Effect patterns (EP) of the climatic variables impacts are controlled by casual models, that is, climatic effect variability model (CEVM) in Iran. This indicates that the observed or hidden variables of the effect patterns (EP) can be defined as either independent variables or dependent variables based on a confirmatory method. However, CPSRM-based modeling in the climatic effect variability (CEV) combines path models and confirmatory factor models. In other words, CPSRM-based models include both climatic hidden and observed variables (Shook et al., 2004; Markus, 2012). A CPSRM-based model takes the relation of the effect direction of data series in the modeling as a relative pattern for the independent variable in period causes dependent variable in the next period. In addition to CPSRM-based model constraints, the direction of their relationship is also revealed by direct or indirect, and total effects (Pakpahan et al., 2017). To depict the directions and their relationship, we use variance and covariance values to predict the causal relations in the model. Furthermore, to define the covariance without causal variability, we use covariance functions. Climatic CPSRM-based modeling is a technique to explore and confirm a climatic series of relationships and to design a measurable pattern for each one based on the covariance among the climatic series (Richter et al., 2016; Javari, 2017d). Climatic CPSRM-based modeling is basically the contemporary scheming of multiple effect pattern based on predicting a series of parameter estimates such as path coefficients, and direct, indirect and total effects analyzing the climatic effect variability (CEV) between the covariance schemed by the theoretical model, and the covariance monitored among the climatic series (Javari, 2017b; Tan et al., 2017). In this study, CPSRM-based different effect techniques, including maximum likelihood (ML), climatic effect patterns (CEP), and first-generation techniques (FGT) have been employed for climatic effect variability (CEV). Maximum likelihood (ML) produces climatic effect patterns (CEP) predicted to expand the possibility that the climatic observed series extract from a set of climatic series with the constructed conceptual model. In this study, the maximum likelihood (ML) is used. Climatic effect patterns (CEP) make direct, indirect and total effects to predict the effect observed patterns from a set climatic series with the considered theoretical model (Newsom, 2015; Temme et al., 2006). Forty years (1975–2014) of estimated climatic data series for Iran were used. A climatic CPSRM-based modeling is represented by two sub-models: (1) the measurement model or external model connecting the manifest variables based on the hidden variables, and (2) the mechanical model or internal model selected based on the relationships between the hidden variables (Villeneuve et al., 2018). To estimate agencies in the CPSRM-based modeling, the examined matrix to be predicted must include estimate means and intercepts (i.e., standardized loadings, score coefficient matrix and the model fitting), either by including an effect direction among variables when the mechanical coefficients are input or by estimating the two types of considered relationships: (l) the mechanical effects of endogenous on other endogenous variables (β); and (2) the mechanical effects of exogenous on endogenous variables (γ) that can be estimated. A general CPSRM-based model estimation (path or mechanical coefficient) is required to predict the effect models with path structures to describe first-generation techniques (FGT). Effect paths (direct, indirect, and total effects) are typically estimated by a general CPSRM-based model based on analyzing effect and causality (PEC). The causal effect paths techniques (CEPT) are defined in three stages and three mechanical equation calculations as follows:

$${\text{y}}_{\text{GEPT}\left(1\right)}={\text{β}}_{01}+{\text{γ}}_{11}{\text{X}}_{1}+{\text{ε}}_{1}$$

$${\text{y}}_{\text{GEPT}\left(2\right)}={\text{β}}_{02}+{\text{β}}_{21}{\text{y}}_{\text{GEPT}\left(1\right)}+{\text{γ}}_{21}{\text{X}}_{1}+{\text{ε}}_{2}$$

$${\text{y}}_{\text{GEPT}\left(2\right)}={\text{β}}_{03}+{\text{β}}_{31}{\text{y}}_{\text{GEPT}\left(1\right)}+{\text{β}}_{32}{\text{y}}_{\text{GEPT}\left(2\right)}+{\text{γ}}_{31}{\text{X}}_{1}+{\text{ε}}_{3}$$

where ${\text{β}}_{\text{i}}$ shows the mechanical intercept value related to each endogenous variable (${\text{y}}_{\text{GEPT}\left(\text{i}\right)}$), ${\text{β}}_{\text{ii}}$ is the path coefficient in the endogenous variable (${\text{y}}_{\text{GEPT}\left(\text{i}\right)}$), on endogenous variables, and ${\text{γ}}_{\text{ii}}$ is the path coefficient in the endogenous variable (${\text{y}}_{\text{GEPT}\left(\text{i}\right)}$) on exogenous (X_j_) variables, related to an endogenous variable, respectively. In this study, CPSRM-based modeling was organized in four causal effect patterns in Iran. The effect patterns in Iran included path analytical models (PAM), factor analysis models (FAM), mechanical analytical models (MAM), and hidden analytical model (HAM). The four effect patterns in Iran included temperature variables effect on precipitation pattern, humidity variables on precipitation pattern, pressures variables on precipitation pattern, and climatic variables of precipitation pattern in Iran. The path analytical model (PAM) is a paradigm based on assessing the climatic variability, thereby representing the direct measurement of a climatic observed variable, and indirect measurement of a climatic hidden variable (i.e., the original hypothesis) in a long-term series (Javari, 2015; Drton and Maathuis, 2017). Hidden analytical model (HAM) is a technique based on analyzing the climatic variability, thereby representing the pattern of path constitution among the climatic hidden variables; it includes both a measurement model and a mechanical model: the measurement model describing the relations between the climatic hidden variables and their observed measures, and the mechanical model showing the relations among the climatic hidden variables themselves (Tenenhaus et al., 2005; Drton and Maathuis, 2017). Factor analysis models (FAM) are the oldest and best-known statistical technique based on investigating relations between sets of climatic observed and hidden variables as a technique constructed for the condition analysis of the unknown relations between the climatic observed and hidden variables. In addition, confirmatory factor analysis (CFA) as a method is constructed for condition analysis of the relationships of the primary hidden variable structure (Kline, 2015). To calculate four generalized effect patterns (GEP) for each climatic series, the selective data were assessed with the goodness of fit indices in Iran.

#### 2.2.4 PLS- based modeling

The PLS-algorithms-based modeling (PABM) classically as a causal method (analysis of the covariance matrix of the contents of the manifest variables the “effect project”, a factor analysis type of the model) (Krepper et al., 2017), was developed by Wold suggested (in 1984) (Wold et al., 1984) for climatic effect patterns analysis (CEPA). Manifest variables are observable variables constructed to transfer data series on the performance of hidden variables, being essential for the climatic effect. In this study, climatic effect factor models (CEFM) (Hanisch, 2017) are the causal method most commonly employed to analyze the second-generation techniques (SGT) between climatic hidden and observed variables. To present the PLS-algorithms-based modeling, we represent climatic construction and estimation to predict the validation and interpretation of factor loadings and climatic hidden variables in climatic-formed PLS in Iran. The effect project of the explanatory patterns is a combination of climatic effect patterns (CEP), and PLS-algorithms-based modeling varies for GIS-based PLS-CPSRM project of each of the climatic series in the optimization methods for high-dimensional patterns (Javari, 2017b). The PLS-algorithms-based modeling (PABM) divides a mechanical model (inner model in the perspective of PLS-CPSRM) into the climatic series representing the constructs in which the mechanical model also displays the relationships (paths) between the constructs and the measurement model (outer models in perspective of PLS-CPSRM) of the constructs showing the relationships between the constructs and the observed variables (Javari, 2017g). In this study, the PLS-CPSRM-based modeling includes two different constructed patterns; one for exogenous hidden variables and one for endogenous hidden variables, for each of 24 main manifest variables. The PLS-CPSRM-based modeling, is considered the measurement models of exogenous and endogenous hidden variables and in this causal processes, uses the hybrid model for specific hidden variables. The models are characterized by PLS-CPSRM-based modeling, different effects as a reflective model or formative model for manifest variables to hidden variables. Effect patterns in PLS-CPSRM-based modeling are effectively combined with both reflective measurement and formative measurement (Agarwal and Osiyevskyy, 2017) constructed for the PLS-CPSRM-based project of temporally various data series used to analyze the climatic effect variability (CEV) in Iran. In the PLS-CPSRM-based modeling, mechanical model shows how the hidden variables are related to each other to predict the effect patterns (EP) of utilizing the three models (reflective, formative, and hybrid models) in Iran. In the PLS-CPSRM-based modeling is controlled the hidden variables operate only as independent variables; they are called exogenous hidden variables, and when hidden variables provide only dependent variables, they are called endogenous hidden variables. However, PLS-CPSRM-based model is used for climatic effect variability (CEV) in Iran. The PLS-CPSRM-based model is an ordinary least squares-based method employed to estimate the path relationships in the model for climatic effect variability (CEV) (Hair et al., 2016). Climatic PLS-CPSRM-based modeling (as the variance-based PLS-CPSRM algorithm) is an algorithm to design based on the reflective, formative, and hybrid methods in the climatic series relations and scheming a quantitative pattern for each one based on the variance in the climatic series (Shook et al., 2004; Markus, 2012; Tenenhaus et al., 2005). To estimate three second-generation techniques (SGT), unlike CPSRM-based modeling, for each climatic series, the selective patterns were evaluated with a set of nonparametric valuation conditions (such as bootstrapping and blindfolding) in Iran (Tenenhaus et al., 2005). In this study, the reliability and validity of the construct (Hulland, 1999; Gupta and Arora, 2017) procedures were used to evaluate the three second-generation techniques (SGT) based on the composite reliability (Cataldo et al., 2017), indicator reliability, convergent validity (Mojtahedi and Oo, 2017), discriminant validity indicators for reflective measurement models (Pappu and Quester, 2017), the convergent validity, collinearity among indicators (Low et al., 2017), significance and relevance of outer CHT for formative measurements models (Avkiran, 2017), coefficients of determination (R^2^), predictive relevance (Q2), size and significance of path coefficients (Choshin and Ghaffari, 2017), and effect size functions for mechanical model in the climatic PLS-CPSRM-based modeling in Iran. The PLS-CPSRM-based modeling algorithm is statistically similar to CPSRM-based modeling algorithm, but it includes the original value of a variable and the value predicted by a modeling algorithm. The effect-based modeling algorithm is briefly analyzed: Firstly, the differences between original value of a variable and the value predicted were estimated and considered on different scales temporally and spatially as estimated error. Then, reliability and validity of the construct are employed to estimate the effect-based modeling. According to the internal consistency reliability explanation, using a different indicator of internal consistency reliability, which is suggested as composite reliability (different outer loadings of the observed variables), is presented as follows:

$$C_{r}={\left(\sum_{i}SOL_{i}\right)}^{2}/\left[{\left(\sum_{i}SOL_{i}\right)}^{2}+\sum_{i}var\left(\varepsilon_{i}\right)\right]$$

where ${\text{SOL}}_{\text{i}}$ is the standardized outer loading of the observed variable i of a certain construct, $\varepsilon_{i}$ is the measurement error of observed variable i, and $\text{var}\left({\text{ε}}_{\text{i}}\right)$ indicates the variance of the measurement error. The composite reliability varies between 0 and 1 so that greater values reveal upper levels of validity. In addition, composite reliability rates of 0.60 to 0. 70 are suitable in exploratory patterns, whereas in climatic CPSRM, rates between 0. 70 and 0.90 can be interpreted as acceptable. The convergent validity index is statistically a quantity positively correlated with different measures of the similar structure (as an index for the reflective model). In this study, estimating convergent validity is considered the average variance extracted (AVE) for situation analysis of the reliability (as an indicator reliability based on outer loadings of the indicators). This also indicates that the variance shared between the construct and its indicator is larger than the measurement error variance. The convergent validity is used as the variance distributed between the construct and its index that if convergent validity is larger than the measurement error variance (AVE value of 0.50 or higher) can be interpreted satisfactory. The discriminant validity index is statistically a unique construct, capturing phenomena with a different structure. In this study, to calculate discriminant validity, the cross-loadings of the indicators and Fornell-Larcker criterion are examined (in the reflective and formative models) to analyze the reliability (it evaluates the square root of the AVE values with the hidden variable correlations). The square root of each construct's AVE should be larger than its maximum correlation with any other structure. To evaluate the SGT models, the PLS-CPSRM algorithms constructed in SmartPLS software reflectively, formative, and hybrid constructs are used (redundancy analysis). Furthermore, SGT models are used to analyze more than two indicators of climatic data series (multicollinearity), and then outer CHT analysis (OWA) is applied to evaluate SGT models (as a method of a linear pattern of the indicator scores and the outer CHT). The values of the outer CHT are employed to analyze the indicator's relative contribution. The next step in PLS-CPSRM algorithms analysis, application of the PLS-CPSRM path patterns to obtain coefficients forming bootstrap distribution, bootstrap samples is determined. The bootstrap method provides certain statistical analysis of the hypothesis with keeping specific outer CHT. Next, bootstrap the Student's t-test is used to calculate the significant series as follows:

$$t_{b}=w_{\left(first\right)1}/bs_{w_{\left(first\right)1}}$$

where $w_{\left(first\right)1}$ is the CHT estimated from the primary model using the primary data samples, and $bs_{w_{\left(first\right)1}}$ is the bootstrap standard error of $w_{\left(first\right)1}$. Hence, when the size of the estimated t value is above 1.96, we can suppose that the path coefficient is significant at a significance level of 5% (two-tailed analyze). An essential point in employing bootstrapping in PLS-CPSRM algorithms is that the signs of the hidden variable scores are unknown. The sign changes the mean value of bootstrap result toward zero, and decreases the corresponding bootstrap standard error, thereby decreasing the t value. The results of three decisions revealed that three types sign changes were suggested: 1) accepting the negative impact of sign changes on the results for the t value, 2) being consistent with the signs in the primary series to avoid sign change-related efforts, and 3) simultaneously comparing the signs of the primary estimation with predicted samples by bootstrap method. This study uses bootstrap confidence interval presenting supplementary evidence on stability of the coefficient evaluation. Evaluation of mechanical model is analyzed as the explained variance of the endogenous hidden variable(s) occurring in a PLS-CPSRM algorithm fit project. In evaluating mechanical model, based on fitting the mechanical model in PLS-CPSRM algorithms, exploratory indicators by the model's predictive abilities are used. The essential indicators to evaluate the mechanical model of the PLS-CPSRM algorithms are the significance of the path coefficients, the level of the coefficient of determination (R^2^) amounts (measuring the model's predictive accuracy), the effect size (impact on the endogenous constructs), the predictive relevance (Q^2^) (examining Stone-Geisser's Q^2^ value) as the indicator of the model's predictive relevance and the total effect size (direct effect and indirect effects).

#### 2.2.5 GIS-based modeling

GIS-based modeling has become an important tool in climatic CPSRM and climate changes whose application in climatology is insufficient (Suryabhagavan, 2017; Javari, 2017e; Bede-Fazekas et al., 2015; García-Gil et al., 2015). Application of GIS modeling methods such as spatial and geostatistical techniques in the ArcGIS is necessary for spatial regionalization (Javari, 2016a; Çelik, 2015; Cheng et al., 2013; El Osta and Masoud, 2015; Feizizadeh et al., 2014). In this study, to analyze the GIS-based effect modeling over 40-years (1975–2014) in the third-generation techniques (TGT), and causal hybrid techniques (CHT) in Iran are employed; (1) development of the GIS-based climatic effect conceptual model; (2) application of the GIS-based climatic effect statistical model at zonal and regional scales; (3) assess and prediction of GIS-based climatic effect results and (4) process and prediction of Comprehensive -based climatic effect results and classification (content-based data classification). Spatial variability of the CPSRM-PLS-based effect of climatic series over the study area was depicted using a set of patterns developed by Comprehensive project. The Comprehensive project focuses on climatic effect patterns such as impacts of the temperature indicators on rainfall, the impacts of the humidity indicators on rainfall, and the impacts of the pressures indicators on rainfall spatially and temporally in Iran. Temporal variability in Comprehensive project for the 170 stations was analyzed based on extracted series in the CPSRM-based effect modeling phase and PLS-based effect modeling phase in the GIS-based effect modeling phase using descriptive statistics such as coefficient of variation (CV%). To analyze the spatial variability of effect and causality (PEC) for distribution of patterns analyzed in the study area, geostatistical spatial statistics techniques were used. Various techniques have been applied to analyze effect and causality (PEC) such as spatial autocorrelation or Global Moran's I statistic (measures spatial autocorrelation based on both spatial distribution of patterns and distribution values of patterns simultaneously), Anselin Local Moran's I Index (classifies spatial clusters of effect patterns with high or low values), Getis-Ord Gi* statistic (recognizes statistically significant spatial clusters of high values (hot spots) and low values (cold spots), as well as Geographically Weighted Regression (GWR) for effect local analysis of linear patterns used to spatially model varying relationships (GWR creates linear patterns based on spatially varying relationships by combining the dependent and explanatory variables of climatic series distributed in the bandwidth of each effect pattern) (Javari, 2017f; ESRI, 2017). The effect pattern and the bandwidth size are dependently controlled using kernel type, bandwidth method, and distance, and the number of neighbor's factors. In all these techniques, climatic series effect on precipitation patterns is examined spatially and temporally. In addition, spatial analytical techniques such as query, overlay, map algebra, and neighborhood representation using the AecGIS_10.5_ software have been employed to analyze effect. In addition, the goodness-of-fit indexes were used to compare two or more causal models such as the chi-square index (X^2^), the goodness-of-fit index (GFI), the adjusted goodness-of-fit index (AGFI) and root mean squared residual (RMR) as AFI, the Tucker–Lewis index (TLI), the Bentler–Bonnet index (BBI), the comparative fit index (CFI), the relative fit index (RFI), and the incremental fit index (IFI) as RFI, the normed chi-square (NC), parsimony ratio (PRATIO), the parsimonious normed fit index (PNFI), the parsimonious goodness-of-fit index (PGFI), root average squared error of approximation (RMSEA) and normed chi-square (CMIN/DF) as PFI, and Akaike information criterion indices (AIC), Browne–Cudeck criterion (BCC), Bayes information criterion (BIC), consistent version of Akaike information criterion (CAIC), and non-central parameter (NCP).

## 3 Discussion and results

### 3.1 CPSRM-based modeling results

**Fig. 1:**
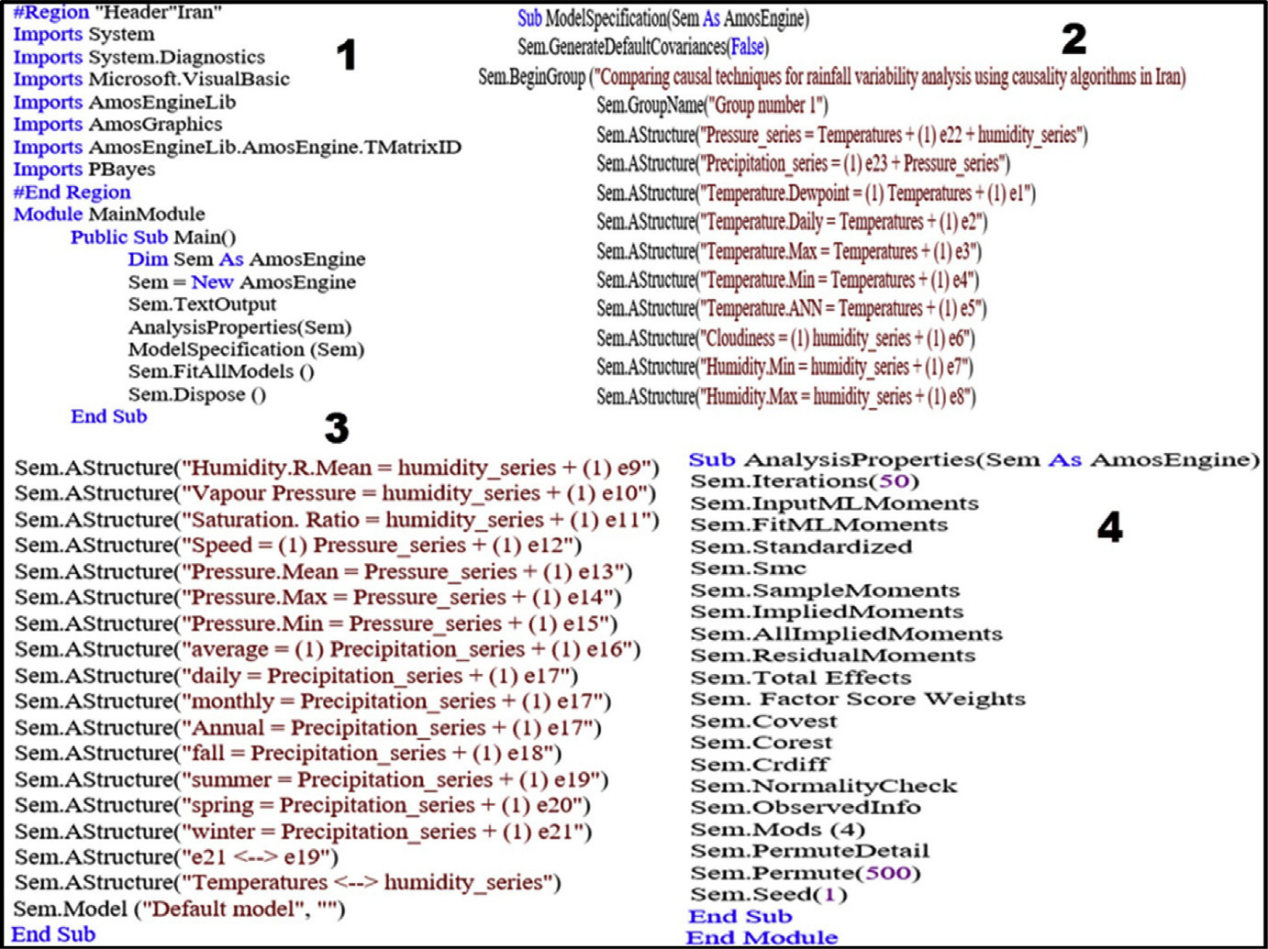
Designed algorithm for rainfall variability analysis.

**Fig. 2:**
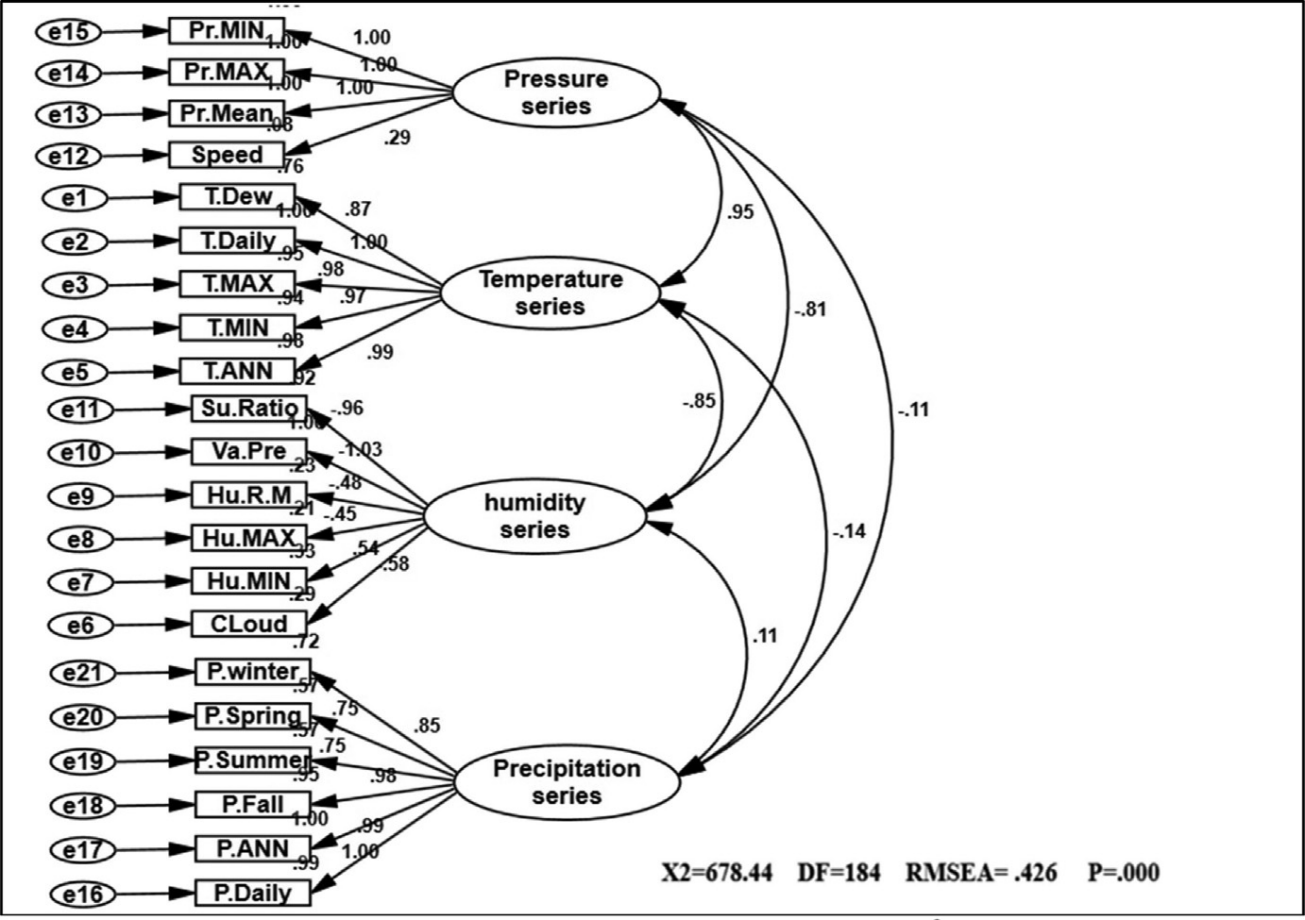
Path diagram of first CPSR model of effectiveness among climatic variables. $\chi^{2}$ = 678.44; P-value of chi-square test = 0.000; goodness-of-fit index = 0.71; root mean square error of approximation = 0.426.

CPSRM-based modeling was applied to covariance-based structure equation model to analyze any probable effect in the climatic series in Iran. This is as a mechanical equation modeling developed to determine relationships between climatic observed series over time (Rahmadi et al., 2017; Javari, 2017b). Direct effects suggest a primary over time, while indirect effects reveal secondary, and total effects show a combination of the direct and indirect effects. Considering CPSRM -based modeling for climate effect in Iran, the study focuses on application of the first-order CFA model constructed to assess the multidimensionality of a constructed algorithm as displayed in Fig. 1. In the CPSRM-based modeling, assessing the hypothesis of self-concept (SC) for a four-factor constitution, is used as a multi-factor construct composed of four factors—precipitation series (PRS), humidity series (HS), pressure series (PS), and temperature series (TS). To analyze the findings of the CPSRM-based modeling, we applied a typical confirmatory factor analysis (CFA) model as a CPSRM-based technique to estimate the relationships between the observed climatic series (Fig. 2). In this example, we investigate the relationship between precipitation series (RPS), humidity series (HS), pressure series (PS), and temperature series (TS) and their reliability. In this regard, we examine whether the four connected patterns of the multi-factor construct, namely climatic twenty-one variables, consider a casual design. Fig. 1 shows the hidden and observed variables based on the constructed algorithm associated with the CPSRM-based modeling to suggest a pattern of the theoretic scheme about a studied climatic series. In general, the relationships between the observed (twenty-one variables) and hidden variables (four variables) are the main aim of the study. These relationships are graphically depicted in path diagram of the CPSRM-based model by two-sided arrows (to analyze the covariance between hidden and observed variables). As Fig. 2 shows, there are twenty-one observed variables in the CPSRM-based model. The observed variables represent twenty-one scale scores extracted from a sample of 170 climatologic and synoptic stations. There are also error terms connected to manifest variables. Each error is the volume of variation in the manifest variable based on measurement error or variation in the conforming hidden factor of the variable CHT.

**Fig. 3:**
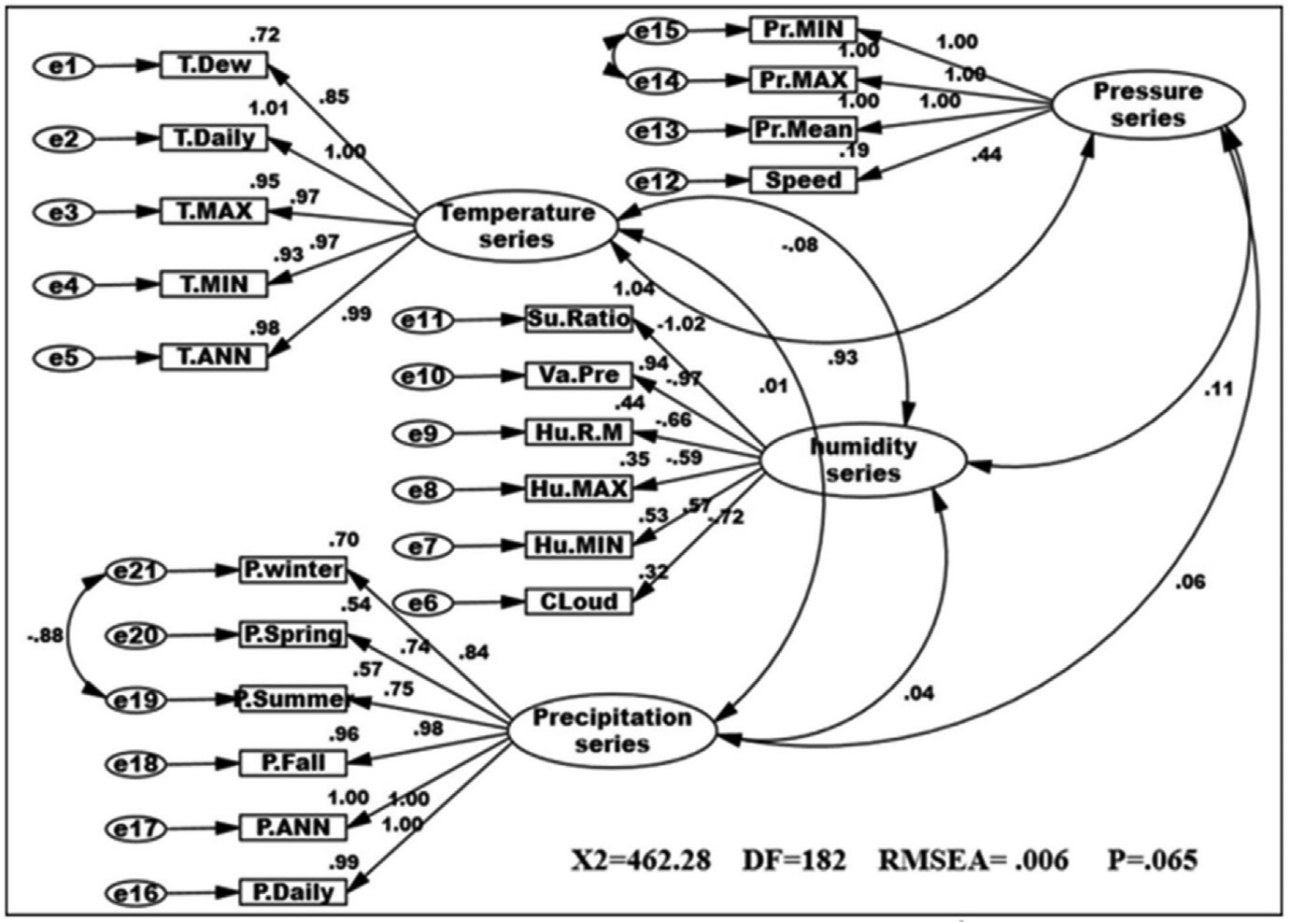
Path diagram of modified model of effectiveness among climatic variables. $\chi^{2}$ = 462.28; P-value of chi-square test = 0.065; goodness-of-fit index = 0.921; root mean square error of approximation = 0.006.

**Fig. 4:**
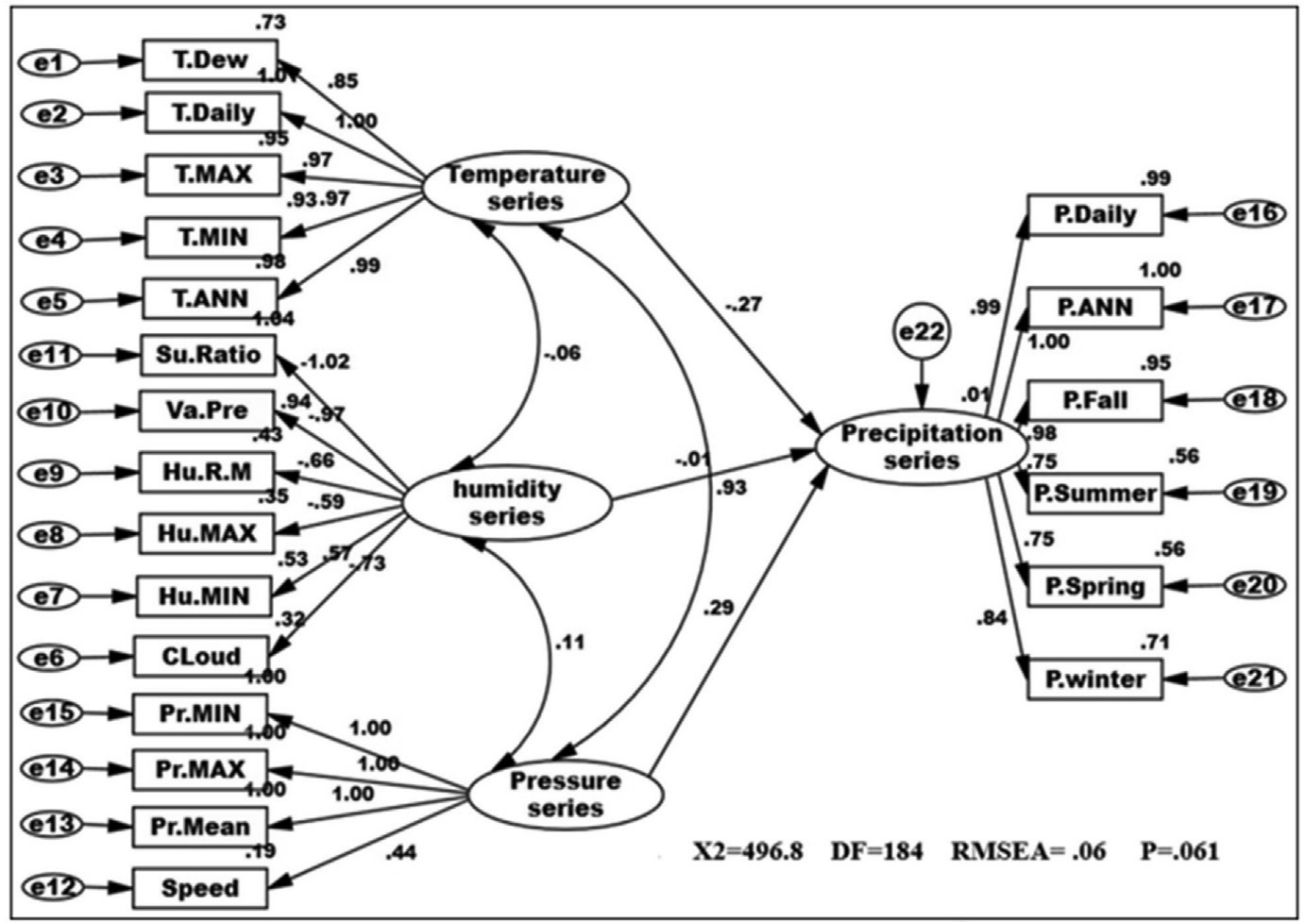
Path diagram of effectiveness among climatic variables on precipitation. $\chi^{2}$ = 496. 8; P-value of chi-square test = 0.061; goodness-of-fit index = 0.901; root mean square error of approximation = 0.06.

In CPSRM is important the mechanical model. Fig. 2 shows the mechanical model examined and the pattern amounts as well as R^2^ for the endogenous variables. Interfaces among and between climatic factors and elements often depend on effect models. Rate of climatic variables are often used to describe that effect models, and to simulation how it changes climatic systems and their interactions. Table 1 summarize the effects of climatic variables such as temperature, pressure, humidity and precipitation shifts in CPSRM based on simulated effect flows by path diagram of CPSR model. The most important result is that climatic variables effects stimulate climate change, in the type of variations in climatic variables, and variables effects varied effect patterns. The CPSR model, the dominant effect, ships less under the CPSR model between temperature and precipitation variables (Fig. 2). In addition, we check the constructed model using goodness of fit indicators ($\chi^{2}$ = 678.44, DF = 184, RMSEA = 0.426, and P = 0.000). The results of the initial fit indicators CFA showed an unacceptable condition of fit (P = 0.000 value is smaller than probability level, 0.05). The model fit indices similar to the comparative fit index (CFI), the goodness-of-fit index (GFI), normed fit index (NFI), Tucker–Lewis index (TLI), and root mean square of error approximation (RMSEA) were designated to assess the model fit (Hair et al., 2016). The results for fitting the series, namely CFI, GFI, NFI, and TLI are less than 0.9, and the RMSEA value is higher than 0.08. Fig. 3 shows the modified model of effect among climatic variables. All goodness of fit statistics are acceptable based on the fit conditions, as the P-values of the chi-square test indicated: >0.05, CFI > 0.90, GFI > 0.90, AGFI > 0.90, and RMSEA < 0.05 ($\chi^{2}$ = 462.28; DF = 182 P-value of chi-square test = 0.065; goodness-of-fit index = 0.921; root mean square error of approximation = 0.006). Furthermore, this CPSRM climatologically studies the impact of climatic series (temperature series, humidity series, and pressure series) on precipitation changes. The results after functioning CPSRM reveal that the CPSRM hypotheses constructed for this study are causally adopted. Fig. 4 shows the model of effect among climatic variables on precipitation. All goodness of fit statistics are acceptable based on the fit conditions, as the P-values of chi-square test indicated: >0.05, CFI > 0.90, GFI > 0.90, AGFI > 0.90, and RMSEA < 0.05 ($\chi^{2}$ = 496.8; DF = 184 P-value of chi-square test = 0.061; goodness-of-fit index = 0.901; root mean square error of approximation = 0.06). The findings not only revealed that these climatic factors had to be well measured, but also indicated that climatic variables effect had to be determined by temporal effect patterns on precipitation variability. Fig. 4 shows a correlation between precipitation series and pressure series. The pressure series factor in this study is defined by pressure maximum, pressure minimum, pressure mean, and wind speed distribution, which can impact the precipitation in Iran. This suggests that pressure series has a suitable and probable impact on precipitation variability. Furthermore, this analysis revealed that there was a relationship between humidity series and precipitation series as a second factor in comparison to pressure factor (Fig. 4). The humidity series factor in this analysis is described by saturation ratio, water vapor pressure, relative humidity mean, relative humidity maximum, relative humidity minimum, and cloudiness values impacting the precipitation in Iran (Fig. 4). This study expanded the effect patterns by designating that there were relationships between climatic series and environmental processes. To predict the climatic variability, accurate selection of effect variability models is highly important. In addition, our results showed that impact of the humidity distribution increased the relative increase rate in the precipitation variables distribution. We also recognized that humidity distribution increased the daily and annual precipitation variables (Fig. 4).

**Table 1:** Statistics properties of the selective series.

Variable	N	Mean	St Dev	CV	Minimum	Median	Maximum	Range	IQR
Elevation	170	1092.3	677.0	61.98	−23.6	1225.1	2465.2	2488.8	1043.8
Daily average rainfall	170	0.9039	0.7483	82.78	0.1332	0.7361	5.0421	4.9090	0.6433
JAN. rainfall	170	50.50	36.72	72.72	9.00	37.50	218.40	209.40	39.39
FEB. rainfall	170	41.24	30.93	75.00	5.70	33.05	232.00	226.30	29.18
MAR. rainfall	170	50.49	32.54	64.45	10.20	43.85	275.80	265.60	36.00
APR. rainfall	170	36.62	26.37	72.02	0.80	33.15	162.50	161.70	38.17
MAY. rainfall	170	18.64	17.44	93.60	0.00	13.30	82.50	82.50	26.35
JUNE. rainfall	170	5.976	10.769	180.22	0.000	1.750	57.900	57.900	4.775
JULY. rainfall	170	4.367	8.381	191.93	0.000	1.300	46.700	46.700	3.400
AUG. rainfall	170	4.98	14.58	292.95	0.00	0.70	111.90	111.90	2.32
SEP. rainfall	170	9.53	35.66	374.30	0.00	0.90	271.50	271.50	2.00
OCT. rainfall	170	20.37	45.36	222.64	0.20	6.40	326.00	325.80	19.00
NOV. rainfall	170	36.51	42.10	115.31	1.40	27.65	300.20	298.80	38.17
DEC. rainfall	170	49.02	39.62	80.82	5.10	35.30	238.20	233.10	44.10
Annual rainfall	170	328.0	271.8	82.87	51.3	267.9	1830.5	1779.2	245.0
Winter rainfall	170	142.01	96.19	67.74	29.00	117.30	726.20	697.20	105.82
Spring rainfall	170	61.24	47.36	77.33	0.80	49.40	206.30	205.50	72.44
Summer rainfall	170	18.87	57.72	305.81	0.00	2.95	429.40	429.40	7.43
Fall rainfall	170	105.89	115.53	109.11	7.90	77.24	843.50	835.60	89.41
Tem. ANN	170	19.429	7.694	39.60	6.000	24.500	29.000	23.000	15.250
Tem. Min	170	12.588	7.454	59.22	2.000	16.000	26.000	24.000	12.000
Tem. Max	170	25.241	7.989	31.65	10.000	29.500	35.000	25.000	15.250
Tem. Daily	170	18.871	7.494	39.71	6.000	23.500	28.000	22.000	14.000
Tem. Dew point	170	5.282	8.163	154.53	−6.000	6.000	22.000	28.000	13.000
Su Ratio	170	7.353	3.951	53.74	3.000	6.500	18.000	15.000	4.000
Vapour Pre	170	10.976	6.672	60.79	4.000	10.500	28.000	24.000	8.000
Humidity R Mean	170	47.176	10.801	22.89	30.000	44.000	71.000	41.000	16.250
Humidity Max	170	64.565	9.764	15.12	47.000	62.500	85.000	38.000	13.500
Humidity Min	170	32.947	11.972	36.34	16.000	29.000	63.000	47.000	16.000
Speed	170	5.259	1.432	27.23	2.000	5.000	10.000	8.000	2.000
Cloudiness	170	39.70	21.28	53.61	3.00	34.00	105.00	102.00	27.25
Pressure Mean	170	906.21	106.25	11.72	757.00	1006.00	1011.00	254.00	210.00
Pressure Max	170	920.32	109.81	11.93	766.00	1022.00	1032.00	266.00	217.00
Pressure Min	170	890.56	104.00	11.68	740.00	986.50	994.00	254.00	203.00

**Fig. 5:**
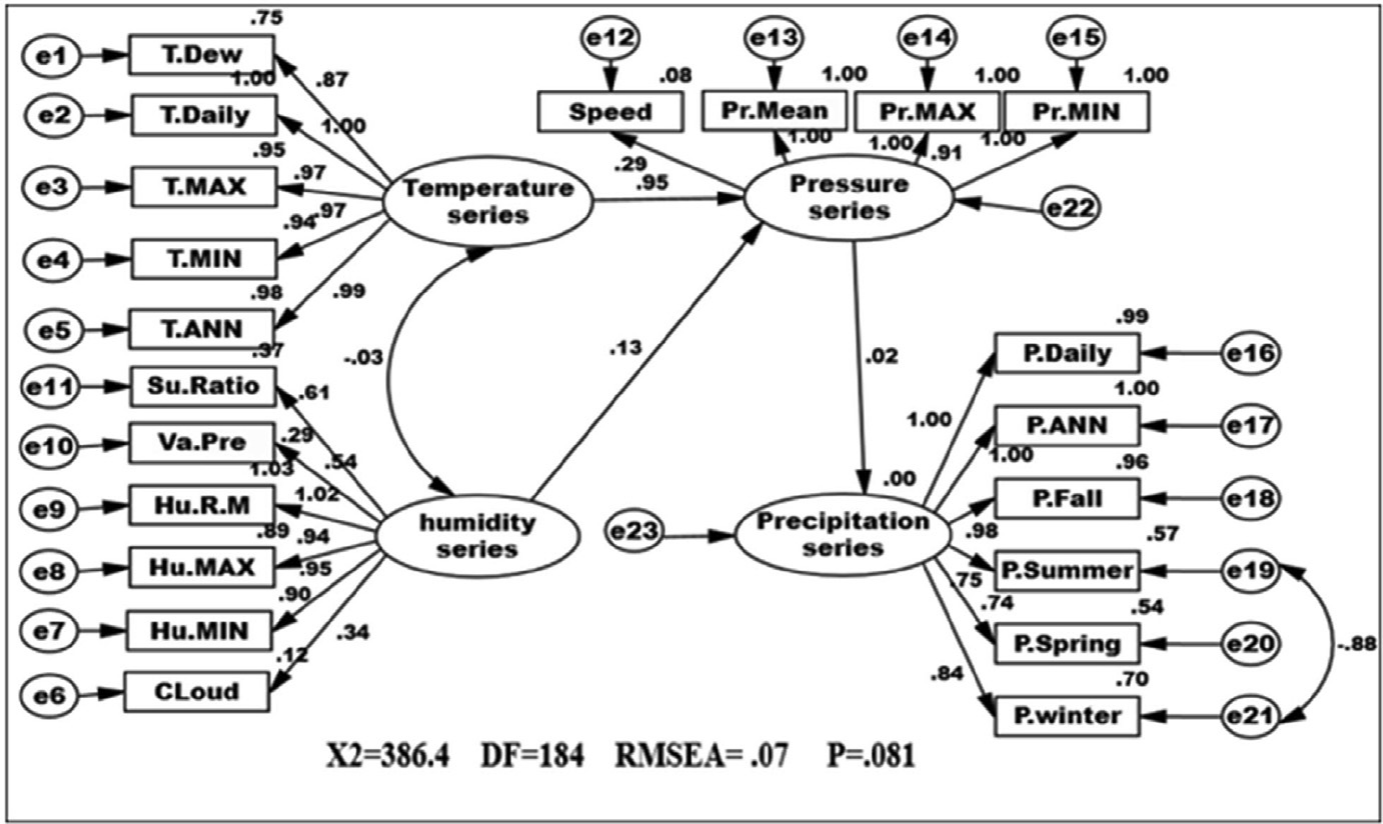
Path diagram of effectiveness among climatic variables on precipitation. $\chi^{2}$ = 386.4; P-value of chi-square test = 0.081; goodness-of-fit index = 0.904; root mean square error of approximation = 0.07 using SRM.

**Fig. 6:**
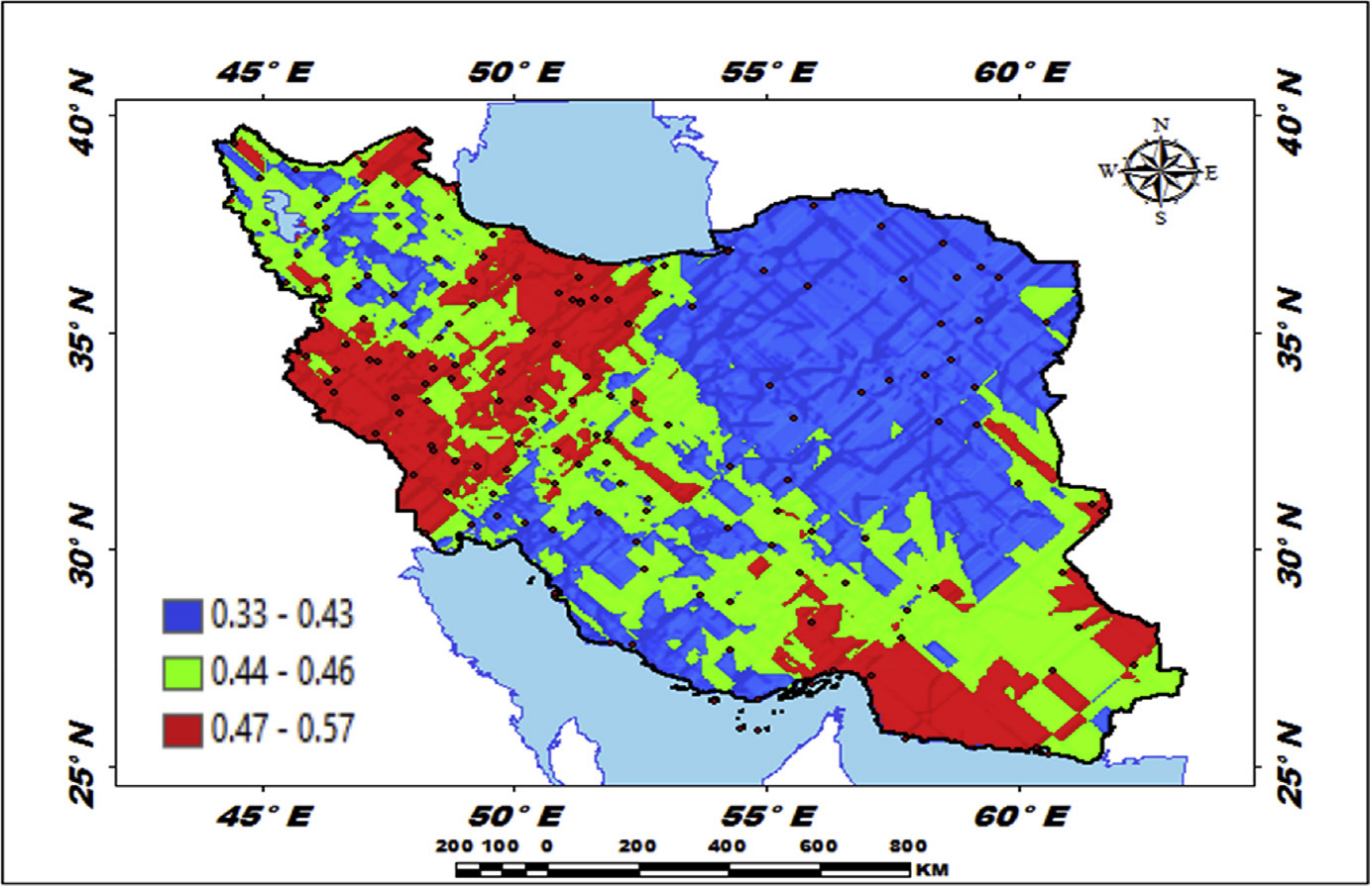
Effectiveness distribution of the pressure series on precipitation series in Iran.

**Fig. 7:**
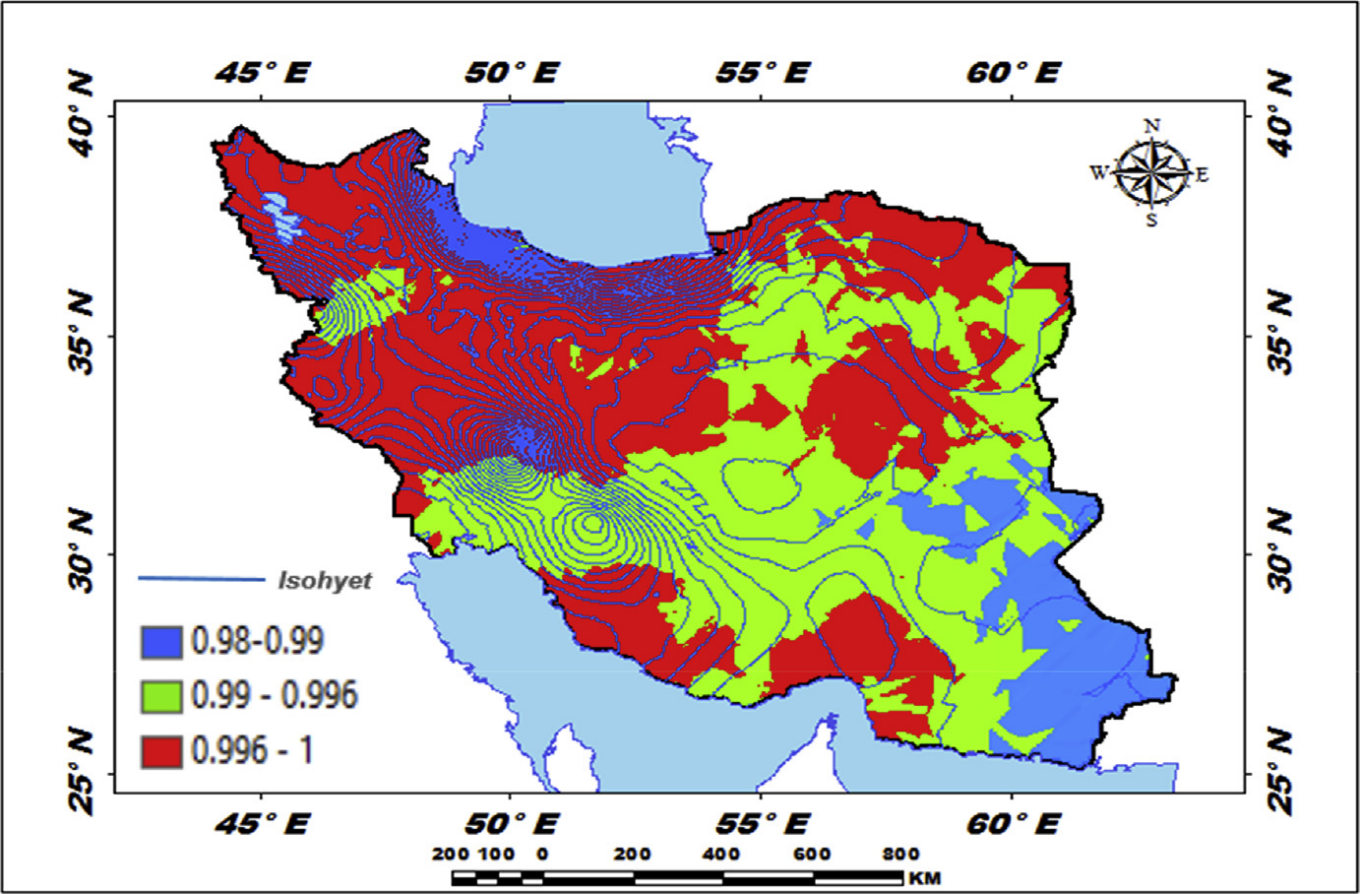
Effectiveness distribution of the minimum pressure series on precipitation series in Iran.

**Fig. 8:**
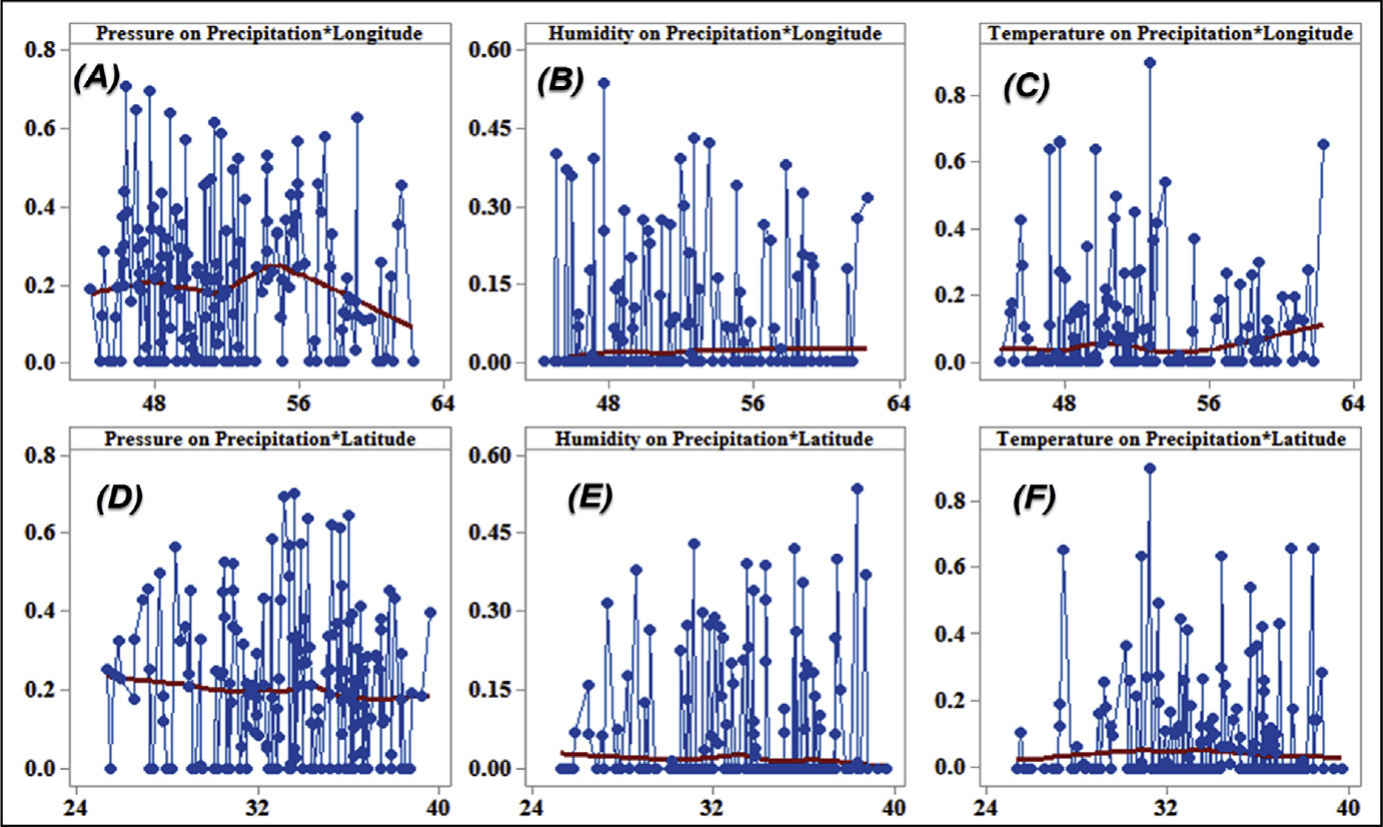
Effectiveness of the climatic series (pressure series, humidity series, and temperature series) on daily precipitation based on longitude and latitude distribution using locally weighted scatterplot smoothing (LOWESS) in Iran.

**Fig. 9:**
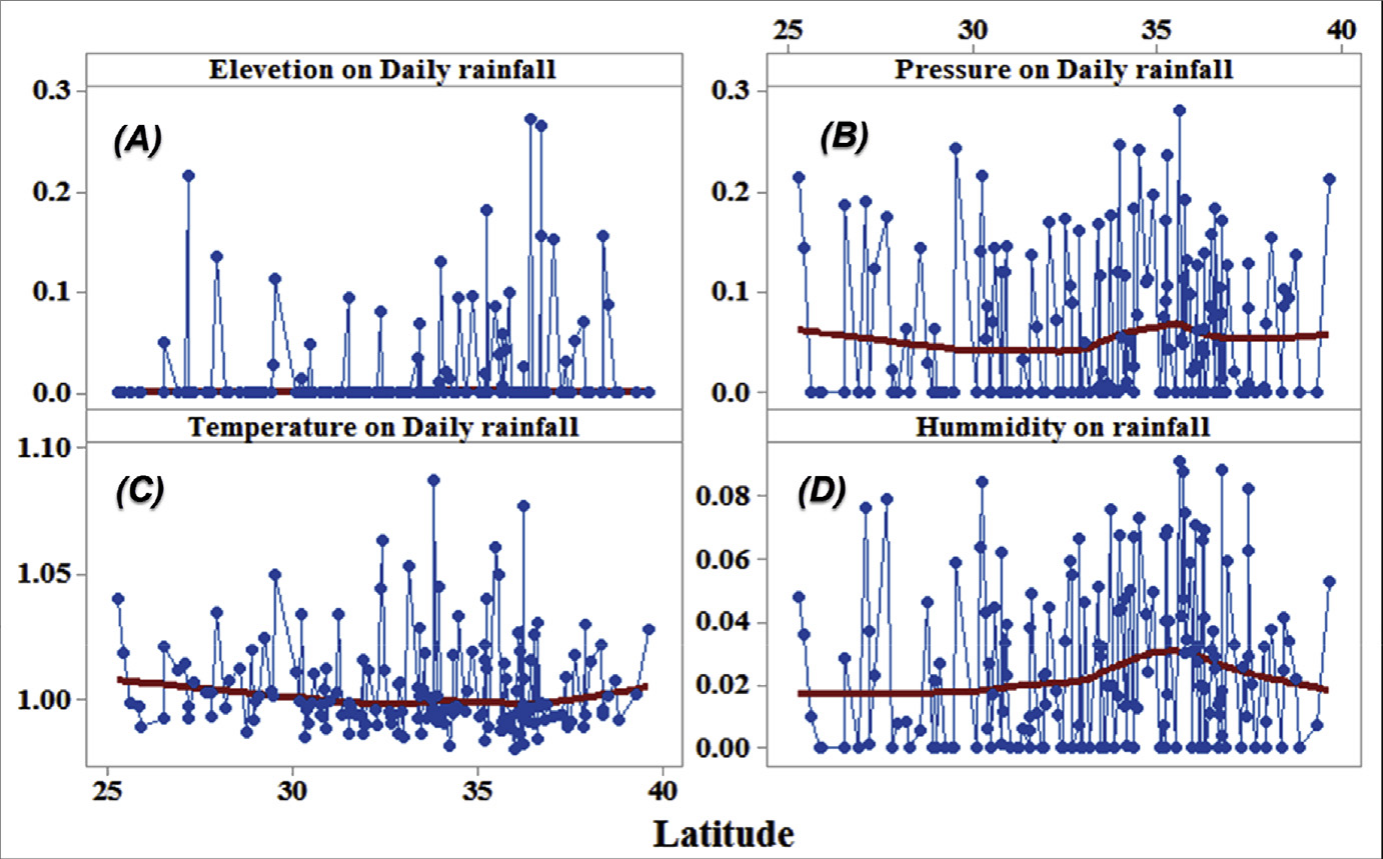
Effectiveness of the climatic series (pressure series, humidity series, elevation, and temperature series) on daily precipitation using locally weighted scatterplot smoothing (LOWESS) in Iran.

Fig. 5 shows that a type of mechanical regression model (MRM) is used in exploratory mechanical equation modeling (EMEM). The temperature series factor in this study is defined by pressure series and pressure series distribution impacting precipitation in Iran. We used EMEM to analyze the single relationships between the temperature, humidity, pressure factors and their effects on precipitation factor (See Fig. 5). The path model fit the data series well, $\chi^{2}$ = 386.4; P-value of chi-square test = 0.081; goodness-of-fit index = 0.904; root mean square error of approximation = 0.07 using MRM. The results revealed important specifications: temperature series had a greater impact (β = 0.95) on pressure series. In the pressure series, minimum pressure more effectively predicted the precipitation. In contrast, humidity series had a lower effect on pressure series. The suggestions based on the test statistics are the Critical Ratio (C.R. = 3.928), representing the parameter estimate or regression CHT (0.055) divided by its Standard Error (S.E. = 0.014), being significant at the level of 0.05. There are differences in the regression CHT of variables between the models for all temperature series, pressures series, and precipitation series (p < 0.001). The critical ratio is the significance of path coefficients. If the Critical Ratio (CR) is >1.96 for a regression CHT, that path is significant at the 0.05 level or higher (that is, its estimated path parameter is significant). We found that the temperature factors were more effective than the other factors (maximum pressure and elevation) on precipitation. This result with theoretical suggestions and experiential effort as well as CPSRM-based modeling phase indicates that pressure factors value and distribution represent one of the effect factors on precipitation. Additionally, our findings show important specifications between the pressure series and minimum pressure factor distribution (See Fig. 5). Fig. 6 indicates that effect spatial distribution is used in the CPSRM-based modeling phase. The temperature, humidity, and pressure series factors on precipitation series in this study are analyzed using GIS-based spatial variability modeling in Iran. Fig. 6 shows effect distribution of the pressure series on precipitation series. It shows that the pressure series impact on precipitation series is significantly decreased from west to east and northeast for spatial variations of pressure series on precipitation, revealing that the change point (about 0.57 or 57%) of the CPSRM-based modeling varies and decreases with the decrease of precipitation amounts and increases the spatial variability factors (about 0.33 or 33%) during the period 1975–2014 in Iran. As Fig. 6 shows, the effect spatial variability coefficient between the precipitation series and pressure series amounts for all stations, implying a strong spatial effect between the precipitation series and pressure series in western and southeast regions, whereas in eastern north and western south regions, a weak spatial effect was demonstrated. Moreover, the specific effect spatial pattern between pressure series amounts on precipitation series was observed in Iran. It was found that there were opposite and contrasted effect directions in Iran. Furthermore, a weak spatial effect (northeastern to southwest trend) and a strong spatial effect (northwest to southeast trend) significance was noticed between the pressure series amounts on precipitation series (Fig. 6). However, spatially, the highest amounts of effect were in the west and southeast for the precipitation and pressure series; and the lowest amounts of effect were in the west and southwest in northeastern in Iran. In Iran, the effect distribution of the minimum pressure series on precipitation series predicted by the GIS-based CPSRM variability ranged from 0.98 to 1 for the causal scenarios (Fig. 7), with a maximum distribution for the western and northern regions, the most important wet area of Iran. All western and northern regions showed significant effect increases. Most of the western and northern regions experienced considerable precipitation distribution based on the minimum pressure series on precipitation in Iran. By CPSRM, the area with effect pattern had developed in the southern regions. For all southern regions, the minimum pressure series on precipitation series for the Hormozghan and Bushehr provinces is predicted based on effect simulation in Iran (Fig. 7). Fig. 7 shows a deep understanding of the effect of climatic series on Iran's rainfall. Fig. 7 presents the effect distribution of the minimum pressure series on the precipitation series. It shows that the minimum pressure series impact on precipitation series is significantly reduced from northwest to southeast for spatial variations of minimum pressure series on precipitation, showing that the change point (about 1 or 100%) of the CPSRM-based modeling fluctuates and decrease with the decrease of precipitation amounts and increases the spatial variability factors (about 0.98 or 98%) during the period of 1975–2014 in Iran. However, spatially, the highest amounts of effect were in the north and northwest for the precipitation and minimum pressure series, and the lowest amounts of effect were in the southeast in Iran. The minimum pressure variable with the greatest effect on the precipitation values was mostly the intensities of northern regions (wet climate regions) in Iran. Fig. 8 presents the effect of the pressure series, humidity series, and temperature series on daily precipitation based on longitude and latitude distribution using locally CHT scatterplot smoothing (LOWESS). Fig. 8 shows the diverse effect patterns of the climatic series on daily precipitation in Iran. The estimated results showed that the effect of the pressure series on daily precipitation decreased with longitude totally from 36° to 64° E longitude during the period 1975–2014 in Iran (Fig. 8A). Furthermore, the results showed that the effect of the humidity series on daily precipitation increased with longitude gradually from 36° to 64° E longitude during the period 1975–2014 in Iran (Fig. 8B). Effect of the temperature series on daily precipitation differenced with longitude and increased with longitude spatially from 56° to 64° E longitude (Fig. 8C). The estimated results showed that effect of the pressure series on daily precipitation increased with latitude totally from 24° to 39° N latitude during the period 1975–2014 in Iran (Fig. 8D). In addition, the results showed that effect of the humidity series on daily precipitation decreased with longitude gradually from 24° to 39° N latitude during the period 1975–2014 in Iran (Fig. 8E). The effect of the temperature series on daily precipitation differenced with latitude and increased with latitude especially from 28° to 32° E latitude (Fig. 8F). Fig. 9 shows the total effects (direct and indirect) of hidden variables (temperature, humidity, elevation, and pressure) on the daily precipitation values, while the variables with the different impacts estimated the effect distribution in climatic series to analyze the effect and causality (PEC) in Iran. The predicted results displayed that the effect of the elevation on daily precipitation increased with latitude partly from 34° to 37° N latitude during the period 1975–2014 in Iran (Fig. 9A). Furthermore, the results showed that the effect of the pressure series on daily precipitation increased with latitude gradually from 25° to 39° N latitude during the period 1975–2014 in Iran (Fig. 9B). The effect of the temperature series on daily precipitation decreased totally with latitude (Fig. 9C). The predicted results showed that the effect of the humidity series on daily precipitation increased with latitude totally from 24° to 35° N latitude and decreased with latitude from 25° to 39° N latitude during the period 1975–2014 in Iran (Fig. 9D).

### 3.2 PLS- based modeling results

#### 3.2.1 Reflective -PLS- based modeling results

**Fig. 10:**
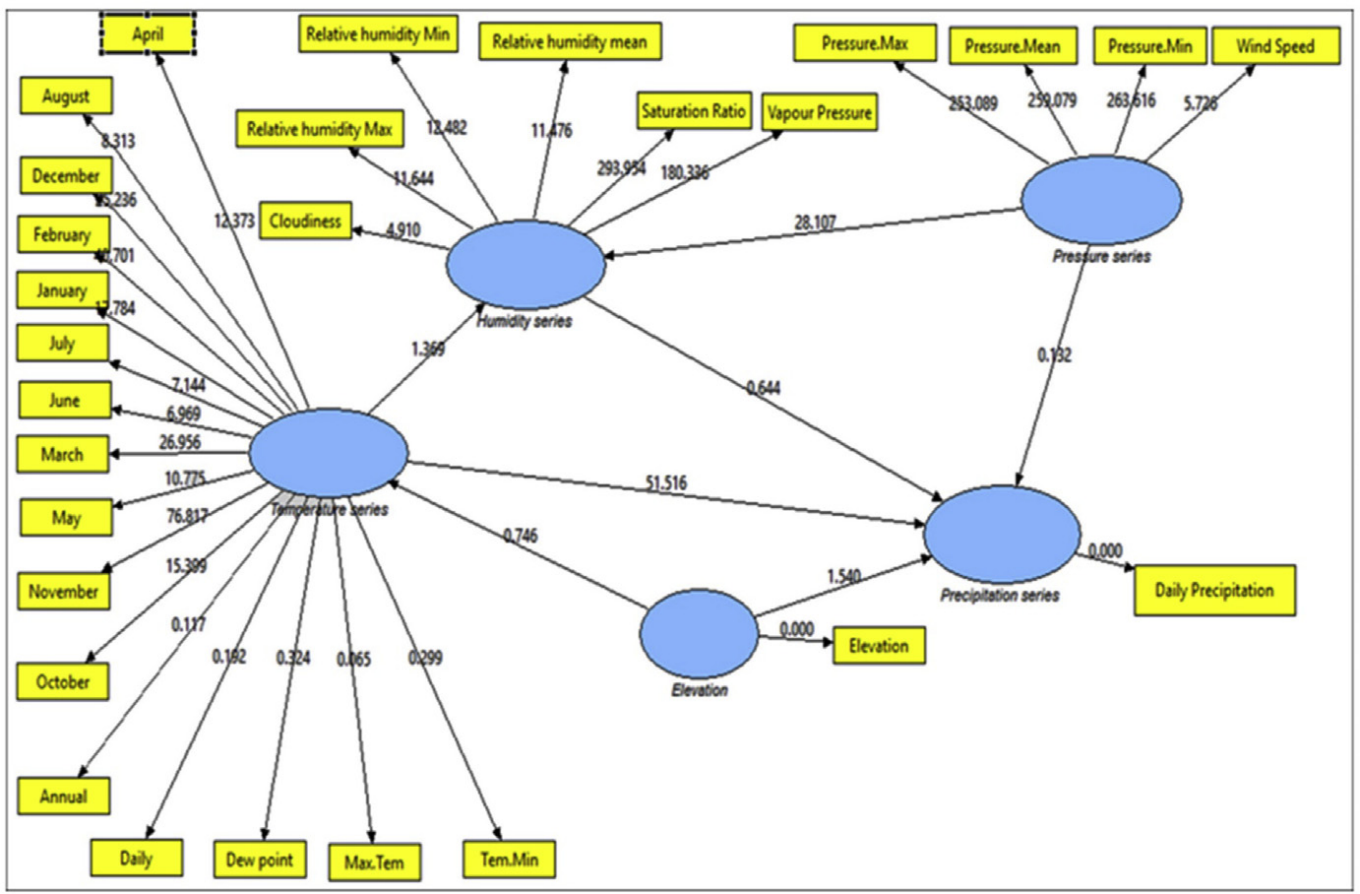
Effectiveness of the climatic series (pressure series, humidity series, and temperature series) on daily precipitation as a reflective model using PLS-algorithms-based modeling in Iran.

In this study, we consider the results from employing the PLS-based modeling techniques (reflectively, formative, and hybrid employed techniques). We describe a summary of the constructed methods and results as well as investigative findings. The main manifest variables of the PLS-CPSRM-based model were studied using the three main models (endogenous construct as reflective measurement model, exogenous constructs as formative measurement model, and hybrid model) as shown in later sections. We use the predicted patterns for all interactions in the measurement models (i.e., the climatic effect loadings and CHT) and the mechanical model (i.e., the climatic effect path coefficients). In the PLS-CPSRM-based modeling, the study focuses on application of the SGT model to assess the multidimensionality of the first model (i.e., reflectively estimated indicators) as shown in Fig. 10. Fig. 10 shows the effect of the climatic series (pressure series, humidity series, and temperature series) on daily precipitation using PLS-algorithms-based modeling in Iran. The effect of the climatic series (pressure series, humidity series, and temperature series) on daily precipitation was analyzed using SmartPLS.2 software. To analyze the effect of the climatic series (pressure series, humidity series, and temperature series) on daily precipitation using PLS-algorithms-based modeling, indicator loadings, Cronbach alphas and composite reliability, convergent validity (AVE), path coefficients, cross-loadings and CHT, correlations, construct scores, hidden variable scores, t-values, and R^2^ values, were used. In this study, the indicator loadings and indicator CHT are estimated to measure the model in the PLS-algorithms-based path model (outer loadings) are used as reflective measurement models, whereas outer CHT are applied in the formative measurement models). Moreover, to examine the results, details are used as the outer CHT, outer loadings, mechanical model path coefficients, and R^2^ amounts. In this study, the systematic analysis of these criteria developed a two-step process, single evaluation of the measurement models and the mechanical model (reliability and validity). Fig. 10 shows the first mechanical model employed for climatic series effect checking. The initial phase in assessing a PLS-algorithms-based path model (a reflective model) is to estimate the outer model to evaluate the measurement model. As Table 2 shows, the outer loadings on the path diagram for reflective model in the PLS-algorithms-based path model. The outer loadings indicator revealed that all loadings were higher than the standard level (0.7), except June temperature, which displayed outer loadings of .6764. Table 3 shows the Student's t-test on the path diagram for reflective model in the PLS-algorithms-based path model. The t-test values revealed that all factor loadings were higher than the critical t values (2.66) for significance levels of 1 % (α = 0.01; two-tailed test), thereby demonstrating validity for all factor loadings. As Table 4 shows, composite reliability varied from .86 to 1.0 for the five constructs more than the standard threshold of 0.7 (composite reliability should be higher than 0.7 (Hair et al., 2016). The average variance extracted (AVE) for the models was above 0.62 for all constructs, while the threshold is .50 (the AVE should be higher than 0.50), as a result, demonstrating convergent validity for all constructs of the PLS-algorithms-based modeling (a reflective model) for climatic series effect in Iran. Furthermore, in evaluating the PLS-algorithms-based path model, the reflective constructs' discriminant validity is used. The typical indicators provided for discriminant validity are the cross-loadings of the indicators and Fornell-Larcker criterion (Hair et al., 2016). Table 5 shows the cross-loadings on the path diagram for reflective model in the PLS-algorithms-based path model. The cross-loadings values revealed that some factor loadings were higher than the critical values (0.1) for significance levels of 1 % (α = 0.01; two-tailed test), thereby demonstrating discriminant validity for some factor loadings. Table 6 shows the Fornell-Larcker indicator (Fornell and Larcker, 1998) on the path diagram for the reflective model in the PLS-algorithms-based path model. Table 6 revealed that all AVEs were greater than the hidden variable correlations. Therefore, discriminant validity was satisfactory with the measurement model of the PLS-algorithms-based modeling. After analyzing the discriminant validity as valid, the next stage is to evaluate the measurement model for distinguish models in the climatic series interactions used the CV-Communality (sum of squared prediction errors for block or SSE/sum of squares of observations for block or SSO that can be also expressed as: I = SSE/SSO) as cross-validated commonality index(I) assessing the quality of the measurement model for each climatic block (Tenenhaus et al., 2005). Table 7 shows the CV-Communality or cross-validated commonality index (I) on the path diagram for reflective model in the PLS-algorithms-based path model. The I index values revealed that all cross-validated communalities were positive, thus demonstrating validity for the measurement model. After validation analysis of the measurement model, the next stage is to evaluate the mechanical model conditions to analyze the climatic series path relationships or the relationships between hidden variables (constructs). The main measures to evaluate the mechanical model in PLS-CPSRM-based modeling are: 1) the estimation of the collinearity (result from when two indicators are much correlated). While more than two indicators are concerned, it is called multicollinearity among sets of constructs, 2) the applicability of the mechanical model relationships and significance of the path coefficients, 3) the estimation of the level of R^2^ values, 4) the evaluation of the effect sizes ($f^{2}$), 5) the evaluation of the predictive relevance ($Q^{2})$ or cross-validated redundancy (CV- Redundancy) and the $q^{2}$ effect sizes (Hair et al., 2016). Before evaluating the mechanical model, we consider the model for collinearity. To consider collinearity, we need to examine each part of predictor structures separately for each subpart of the mechanical model. To evaluate the level of collinearity, we estimated the tolerance. The tolerance (each indicator's tolerance (VIF) value should be between 0.20 and 5) corresponds to the amount of variance of each indicator not described by other indicators in a block (Astrachan et al., 2014). Table 8 shows the indicator's tolerance (VIF) value on the path diagram for the model in the PLS-algorithms-based path model. Table 8 shows that all VIF values are well below the threshold of 5. Therefore, VIF values indicating that the climatic series (temperature, pressure, elevation, and humidity series) are highly inter-correlated and the small changes in the data values may lead to large changes in the estimates of the path coefficients of the PLS-algorithms-based modeling. Before evaluating the significance of the path coefficients, we analyzed the coefficient of determination $\left(R^{2}\right)$, an important indicator of the estimate of the model's predictive precision is based on the squared correlation between an endogenous construct's actual and forecasted values in the PLS-algorithms-based path model. Table 9 shows the coefficient of determination values on the path diagram for the model in the PLS-algorithms-based path model. The $R^{2}$ values ranges from 0.112358 to 0.375019, and higher levels show higher levels of analytical accuracy. Therefore, $R^{2}$ values indicate that the pressure series (37.5%), humidity series (35.7%), elevation series (15.5%), and temperature series (11.2%) are highly efficient in the PLS-algorithms-based modeling, respectively. We analyzed the significance of the path coefficients using the student's t-test value showing the climatic series relationships. Table 10 shows the coefficients and t-test value based on the path diagram for the model in the PLS-algorithms-based path model. Table 10 shows that all path coefficients values are well above the critical t-values (2.58). Therefore, path coefficients and significance level indicating that all four-climatic series effect (temperature, pressure, elevation, and humidity series) on daily precipitation were statistically accepted in the PLS-algorithms-based modeling. After evaluating the path coefficients, we consider the model for total effects. To consider total effects, we need to examine each path of the predictor structures along with using the student's t-test value for total effects. Table 11 shows the total effects and level of significance based on the path diagram for the model in the PLS-algorithms-based path model. Table 11 shows that significance values of the total effects are well above the critical t-values (1.96 and 2.58). Therefore, total effects and significance level indicating that all four-climatic series effect (temperature, pressure, elevation, and humidity series) on daily precipitation were statistically accepted in the PLS-algorithms-based modeling. Finally, after assessing the total effects, we consider the model for the predictive relevance ($Q^{2})$ or cross-validated redundancy (CV- Redundancy). Table 12 shows the predictive relevance ($Q^{2})$ or CV-Redundancy and level of significance based on the path diagram for the model in the PLS-algorithms-based modeling. Table 12 showed that significance values of the predictive relevance ($Q^{2})$ or CV- Redundancy were positive and suitable for the model. Therefore, predictive relevance ($Q^{2})$ or CV-Redundancy and level of significance indicating that all four-climatic series effect (temperature, pressure, elevation, and humidity series) on daily precipitation were statistically suitable and accepted in the PLS-algorithms-based modeling.

**Table 2:** Outer loadings for reflective model in the PLS-SEM.

	Elevation	Humidity series	Precipitation series	Pressure series	Temperature series
April	0.0000	0.0000	0.0000	0.0000	**0.7059**
August	0.0000	0.0000	0.0000	0.0000	**0.7601**
Daily precipitation	0.0000	0.0000	**1.0000**	0.0000	**0.0000**
December	0.0000	0.0000	0.0000	0.0000	**0.8376**
Elevation	**1.0000**	0.0000	0.0000	0.0000	0.0000
February	0.0000	0.0000	0.0000	0.0000	**0.8165**
January	0.0000	0.0000	0.0000	0.0000	**0.7278**
July	0.0000	0.0000	0.0000	0.0000	**0.7134**
June	0.0000	0.0000	0.0000	0.0000	**0.6764**
March	0.0000	0.0000	0.0000	0.0000	**0.8026**
May	0.0000	0.0000	0.0000	0.0000	**0.6195**
November	0.0000	0.0000	0.0000	0.0000	**0.9660**
October	0.0000	0.0000	0.0000	0.0000	**0.8365**
Pressure Max	0.0000	0.0000	0.0000	**0.9999**	0.0000
Pressure mean	0.0000	0.0000	0.0000	**1.0000**	0.0000
Pressure Min	0.0000	0.0000	0.0000	**0.9999**	0.0000
Relative humidity Max	0.0000	**0.8098**	0.0000	0.0000	0.0000
Relative humidity Min	0.0000	**0.8371**	0.0000	0.0000	0.0000
Relative humidity mean	0.0000	**0.8294**	0.0000	0.0000	0.0000
Saturation ratio	0.0000	**0.9701**	0.0000	0.0000	0.0000
Vapor pressure	0.0000	**0.9555**	0.0000	0.0000	0.0000

Bold value signifies the outer loadings should be higher than 0.7.

**Table 3:** t Test for reflective model in the PLS-SEM.

	Elevation	Humidity series	Precipitation series	Pressure series	Temperature series
April					12.06
August					8.32
December					27.85
Elevation					6.67
February					40.84
January					17.32
July					7.29
June					7.14
March					28.76
May					12.52
November					156.07
October					15.23
Pressure Max				122269.74	
Pressure Mean				401154.5	
Pressure Min				91725.7	
Relative humidity Max		16.37			
Relative humidity Min		16.42			
Relative humidity mean		15.62			
Saturation ratio		131.07			
Vapor pressure		110.01

**Table 4:** Evaluation indictors for reflective model in the PLS-SEM.

Climatic series	AVE	Composite reliability	R square	Cronbach's Alpha
Elevation	1	1		1
Humidity series	0.63	0.86	0.47	0.86
Precipitation series	1	1	0.98	1
Pressure series	0.78	0.93		0.88
Temperature series	0.62	0.88	0.46	0.89

**Table 5:** Cross loadings for reflective model in the PLS-SEM.

	Elevation	Humidity series	Precipitation series	Pressure series	Temperature series
April	0.375208	0.005000	0.687212	0.056089	0.705892
August	−0.318292	−0.030500	0.746352	0.036000	0.760114
Daily precipitation	−0.092508	−0.035097	1.000000	0.022069	0.991360
December	−0.117705	−0.107210	0.862806	−0.075828	0.837606
Elevation	1.000000	−0.019793	−0.092508	0.002300	−0.077753
February	0.078707	−0.011427	0.833689	−0.013658	0.816470
January	−0.099963	−0.071362	0.758373	−0.087561	0.727783
July	−0.228073	0.041678	0.673368	0.087886	0.713352
June	−0.209749	0.024882	0.622624	0.122444	0.676371
March	0.180420	0.025945	0.810517	0.023235	0.802560
May	0.244085	0.024085	0.557253	0.159853	0.619474
November	−0.153473	−0.065068	0.966694	0.015313	0.966001
October	−0.270038	−0.065837	0.830143	0.048675	0.836516
Pressure Max	0.002040	0.625861	0.022597	0.999909	0.033739
Pressure Mean	0.003002	0.630166	0.020865	0.999975	0.032140
Pressure Min	0.001856	0.629324	0.022743	0.999879	0.034196
Relative humidity Max	−0.016691	0.809779	−0.048260	0.354291	−0.041641
Relative humidity Min	−0.003028	0.837111	−0.034095	0.159042	−0.041057
Relative humidity mean	0.008748	0.829383	−0.045234	0.213263	−0.046493
Saturation ratio	−0.029236	0.970128	−0.028064	0.698108	−0.027712
Vapor pressure	−0.019474	0.955481	−0.024055	0.759026	−0.021398

**Table 6:** Fornell-Larcker indicator for reflective model in the PLS-SEM.

	Humidity series	Precipitation series	Pressure series	Elevation
Elevation	0.8830			
Humidity series	−0.019793	1		
Precipitation series	−0.092508	−0.035097	0.9999	
Pressure series	0.002300	0.628505	0.022069	0.7747
Temperature series	−0.077753	−0.033492	0.991360	0.03336

**Table 7:** CV-Communality indicators for reflective model in the PLS-SEM.

Total	SSO	SSE	1-SSE/SSO
Elevation	170	170	1
Humidity series	850	276.532171	0.674668
Precipitation series	170	170	1
Pressure series	510	40.240350	0.921097
Temperature series	1870	880.886447	0.528938

**Table 8:** Indicator's tolerance (VIF) for model in the PLS-SEM.

Model	Unstandardized coefficients		Standardized coefficients	t	Sig.	Correlations			Collinearity statistics	
	B	Std. Error	Beta			Zero-order	Partial	Part	Tolerance	VIF
(Constant)	−1.718E-7	0.010		0.000	1.000					
Elevation series	−0.015	0.010	−0.015	−1.507	0.134	−0.093	−0.117	−0.015	0.993	**1.007**
Humidity series	0.008	0.013	0.008	.594	0.554	−0.035	0.046	0.006	0.601	**1.663**
Pressure series	−0.016	0.013	−0.016	−1.215	0.226	0.022	−0.094	−0.012	0.602	**1.662**
Temperature series	0.991	0.010	0.991	97.565	0.000	0.991	0.991	0.985	0.988	**1.013**

Bold indicates each VIF value in between 0.2 and 5.

**Table 9:** R^2^ values for model series in the PLS-SEM based model.

Series	$R^{2}$
Elevation	0.155254
Humidity series	0.357369
Pressure series	0.375019
Temperature series	0.112358

**Table 10:** Results of the significance of the path coefficients based on PLS-SEM model.

	Original sample (O)	Sample mean (M)	Standard deviation (STDEV)	Standard error (STERR)	T = (O)/(STERR)	Result
Elevation → Precipitation series	0.025269	0.016630	0.009274	0.009274	2.72471	Accept***
Humidity series → Precipitation series	0.107726	0.007935	0.021851	0.021851	4.93002	Accept***
Pressure series → Precipitation series	0.075810	0.015902	0.010698	0.010698	7.08637	Accept***
Temperature series → Precipitation series	0.990959	0.991479	0.004110	0.004110	241.10924	Accept***

Critical t-values for a two-tailed test is: <2.58 (P-value = .001***).

**Table 11:** Results of the significance of the total effects based on PLS-SEM model.

	Original sample (O)	Sample mean (M)	Standard deviation (STDEV)	Standard error (STERR)	T statistics (|O/STERR|)	Result
Elevation → Precipitation series	0.192286	0.083945	0.098227	0.098227	1.95757	Accept***
Humidity series → Precipitation series	0.057716	0.008112	0.021184	0.021184	2.72451	Accept***
Pressure series → Precipitation series	0.629831	0.630112	0.023463	0.023463	26.84399	Accept***
Temperature series → Precipitation series	0.990537	0.991181	0.004273	0.004273	231.81842	Accept**^,^ ***

Critical t-values for a two-tailed test is: <2.58 (P-value = .001***) and <1.96 (p = .05**).

**Table 12:** Results of the significance of the CV- Redundancy based on PLS-SEM model.

Total	SSO	SSE	1-SSE/SSO
Elevation series	18.107368	18.000368	0.005909
Humidity series	850.000000	699.045499	0.177594
Precipitation series	170.000000	8.826225	0.948081
Temperature series	1870.000000	1869.145347	0.000457

#### 3.2.2 Formative -PLS- based modeling results

**Fig. 11:**
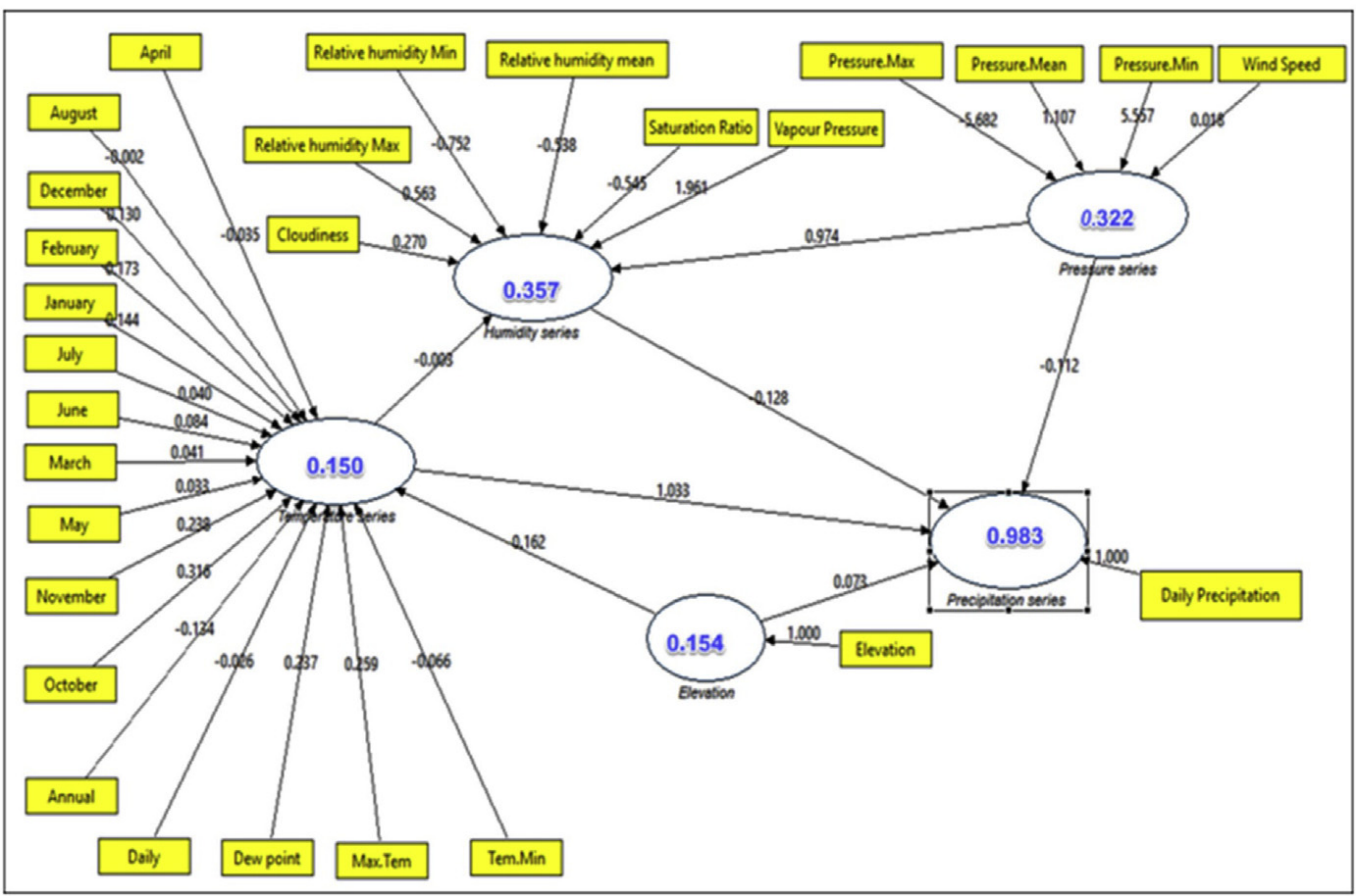
Effectiveness of the climatic series (pressure series, humidity series, and temperature series) on daily precipitation as a formative model using PLS-algorithms-based modeling in Iran.

**Fig. 12:**
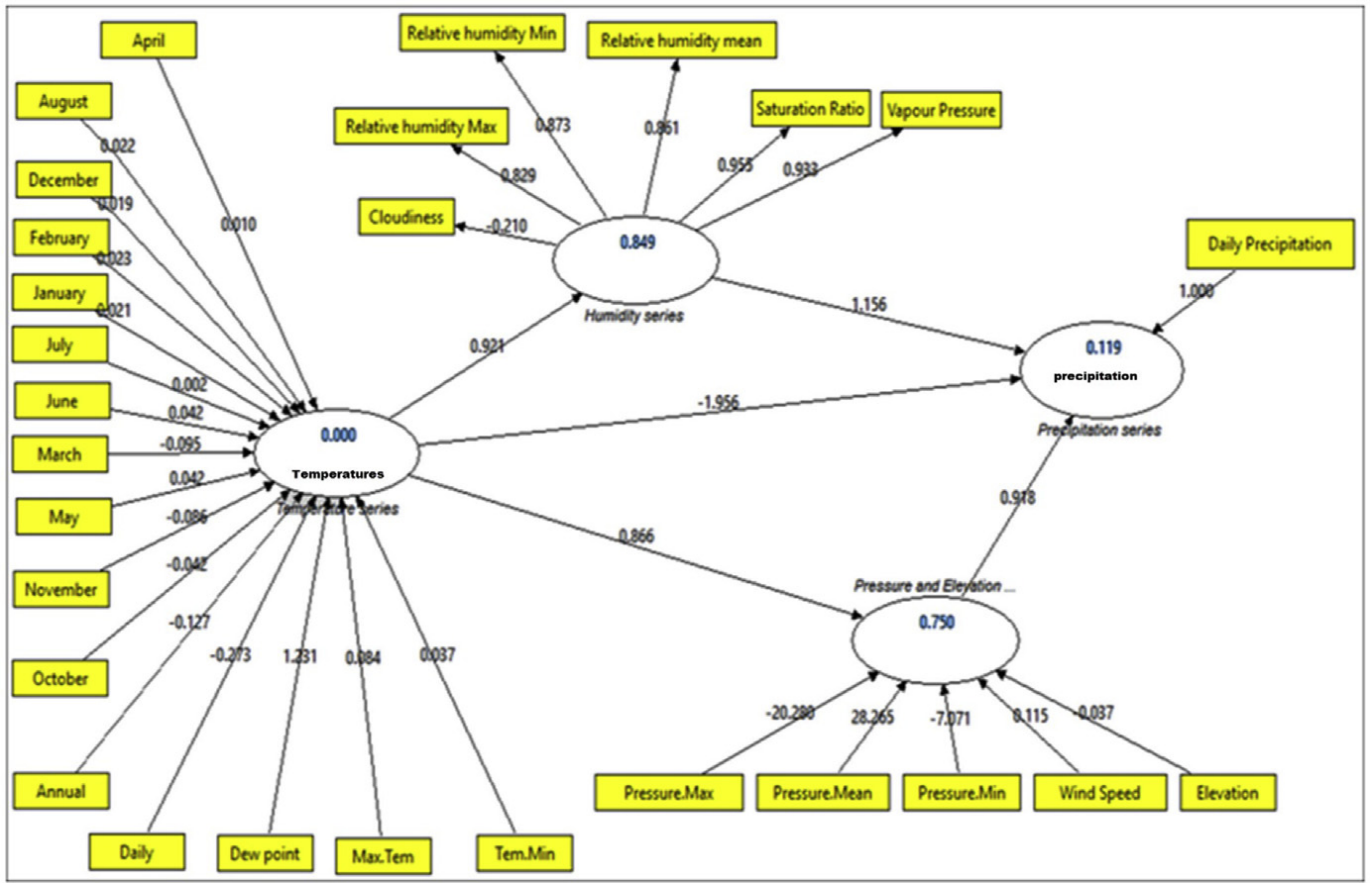
Effectiveness of the climatic series (pressure series, humidity series, and temperature series) on daily precipitation as a hybrid model using PLS-algorithms-based modeling in Iran.

In addition, in this study, we consider the results from employing the PLS-based modeling techniques (formative used techniques) to analyze the second-generation techniques (SGT). We describe a summary of formative methods and results as well as investigative findings. The main manifest variables of the PLS-CPSRM-based model were studied using the main model (endogenous construct as formative measurement model) as shown in Fig. 11. We use the analyzed patterns for all interactions in the measurement models (i.e., the climatic effect loadings and CHT) and the mechanical model (i.e., the climatic effect path coefficients). In the PLS-CPSRM-based modeling, the study focuses on the application of the SGT model to assess multidimensionality of the second model (i.e., formative estimated indicators) as shown in Fig. 11. Fig. 11 shows effect of the climatic series (elevation series, pressure series, humidity series, and temperature series) on daily precipitation using the PLS-algorithms-based modeling in Iran. Effect of the climatic series (elevation series, pressure series, humidity series, and temperature series) on daily precipitation was analyzed using SmartPLS.2 software. To examine effect of the climatic series (elevation series, pressure series, humidity series, and temperature series) on daily precipitation using the PLS-algorithms-based modeling, the indicator loadings, Cronbach alphas and composite reliability, convergent validity (AVE), path coefficients, cross-loadings and CHT, correlations, construct scores, hidden variable scores, t-values, and R^2^ values were applied. Fig. 11 shows the second mechanical model as a formative pattern for climatic series effect checking. The initial phase in assessing a PLS-algorithms-based path model (a formative model) is to assess the outer model to estimate the measurement model. As results show, the outer loadings on the path diagram are suitable for the formative model in the PLS-algorithms-based path model. The outer loadings indicator showed that all loadings were higher than the standard level (0.7), except relative humidity displaying outer loadings of 0.16366. In PLS-based modeling, an effect increase of humidity and pressure series and a decrease in temperature series on precipitation series is estimated in Iran (Fig. 12). The effect of the relative humidity, vapor pressure and saturation ratio will increase up to 0.955 in the PLS-CPSRM. The variability in the precipitation distribution however, is more changeable due to spatiotemporal effect and the variability controlled by the various effect models employed (Fig. 12). Table 13 shows that the student's t-test on the path diagram is suitable for the formative model in the PLS-algorithms-based path model. The t-test values (Student's t-test for outer CHT varied from 3.39 to 4.876 for the five constructs) displayed that all path coefficients were greater than the critical t values (2.66) for significance levels of 1 % (α = 0.01; two-tailed test), thus presenting validity for all outer CHT. The key measures to evaluate the formative model in the PLS-CPSRM-based modeling is estimating the collinearity. To study collinearity, we should examine each part of predictor structures separately for each subpart of the formative model. To estimate the level of collinearity, we estimated the indicator's tolerance (VIF) values $VIF=1/\left(1-R^{2}\right)$. Table 14 shows the indicator's tolerance (VIF) value on the path diagram for the formative model in the PLS-algorithms-based path model. Table 14 shows that all VIF values are well lower than the threshold of 5. Therefore, the VIF values showing that the climatic series (temperature, pressure, elevation, and humidity series) are highly inter-correlated. After analyzing the VIF values as valid, the next stage is to evaluate the formative model to distinguish the suitable models in the climatic series effect is estimating the coefficient of determination (R2) as quality assessment of the formative model for each climatic series. Table 15 shows the coefficient of determination $\left(R^{2}\right)$ on the path diagram for the formative model in the PLS-algorithms-based path model. The $R^{2}$ index values revealed that they varied from 0.983 to 0.322 for the five constructs, thus indicating different validity for the formative model. Therefore, $R^{2}$ values representing the humidity series (35.7%), pressure series (32.2%), elevation series (15.4%), and temperature series (15 %) have different efficiency in the PLS-algorithms-based modeling, respectively. After the $R^{2}$ index analysis of the formative model, the next stage is to assess the mechanical model conditions to analyze the climatic series relationships between the hidden variables (constructs). We analyzed significance of the path coefficients using the student's t-test value showing the climatic series relationships. Results show the coefficients and t-test value based on the path diagram for the model in the PLS-algorithms-based path model. After assessing the path coefficients, we consider the model for total effects. To assess the formative model, the predictive relevance (Qˆ2) or cross-validated redundancy (CV-Redundancy) is used. Table 16 shows the predictive relevance ($Q^{2})$ or CV- Redundancy based on the path diagram for the formative model in the PLS-algorithms-based modeling. Table 16 shows that the significance values of the $Q^{2}$ or CV-Redundancy are positive and suitable for the model. Therefore, the predictive relevance ($Q^{2})$ representing that all four-climatic series effect (temperature, pressure, elevation, and humidity series) on daily precipitation are statistically fitted and accepted in the PLS-algorithms-based for formative modeling.

**Table 13:** Results of the t-statistics based on PLS-SEM formative model.

	Original series (O)	Series mean (M)	Standard deviation (STDEV)	Standard error (STERR)	T statistics (O/STERR)
Temperature series → Daily Precipitation	0.270165	0.266930	0.058780	0.058780	4.596247
Relative humidity series → Daily Precipitation	0.563010	0.566548	0.121053	0.121053	4.650942
Pressure series → Daily Precipitation	5.557346	5.446230	1.637093	1.637093	3.394643
Elevation series → Daily Precipitation	5.057346	5.046230	1.037093	1.037093	4.876463

**Table 14:** Results of the indicator's tolerance (VIF) on PLS-SEM formative model.

	Original series (O)	VIF = 1/(1-R^2^)
Temperature series → Daily Precipitation	0.270165	$\mathrm{VIF}=\frac{1}{1-0.270165^{2}}$ = 1.078
Relative humidity series → Daily Precipitation	0.563010	1.464
Pressure series → Daily Precipitation	5.557346	−0.0334
Elevation series → Daily Precipitation	5.057346	−0.0406

**Table 15:** R^2^ values for PLS-SEM based formative model.

Climatic series	$R^{2}$
Elevation series	0.154
Humidity series	0.357
Precipitation series	0.983
Pressure series	0.322
Temperature series	0.150

**Table 16:** Results of the significance of the CV- Redundancy based on PLS-SEM formative model.

Total	SSO	SSE	1-SSE/SSO
Elevation series	25.610	0.445	0.9826
Humidity series	1020	867.156135	0.1498
Precipitation series	170	69.833451	0.5892
Temperature series	2720	2704.694609	0.0056

#### 3.2.3 Hybrid-PLS- based modeling results

In this study, we consider the results from employing PLS-based modeling techniques to analyze the second-generation techniques (SGT). We use the analyzed patterns for all interactions in the measurement models and the mechanical model. Effect of the climatic series (elevation series, pressure series, humidity series, and temperature series) on daily precipitation was analyzed using hybrid techniques. The initial phase in assessing a PLS-algorithms-based path model (a hybrid model) is to assess the outer model to estimate the measurement model. Table 17 shows the student's t-test on the path diagram for hybrid model in the PLS-algorithms-based path model. The t-test values (Student's t-test for outer CHT varied from 2.32 to 25.57 for the four constructs) displays that all path coefficients are greater than the critical t-values (1.96) for the significance levels of 5% (α = 0.05; two-tailed test), thereby presenting validity for all outer CHT. After assessing the path coefficients, we consider the model for total effects. To assess the hybrid model, the predictive relevance ($Q^{2})$ or cross-validated redundancy (CV-Redundancy) is used. Results show the predictive relevance ($Q^{2})$ or CV-Redundancy based on the path diagram for the hybrid model in the PLS-algorithms-based modeling that the significance values of the $Q^{2}$ or CV-Redundancy are positive and suitable for the model. Therefore, predictive relevance ($Q^{2})$ representing all four-climatic series effect (temperature, pressure, elevation, and humidity series) on daily precipitation are statistically fitted and accepted in the PLS-algorithms-based for hybrid modeling.

**Table 17:** Results of the t-statistics based on PLS-SEM hybrid model.

	Original sample (O)	Sample mean (M)	Standard deviation (STDEV)	Standard error (STERR)	T statistics (|O/STERR|)
Humidity series → Precipitation series	2.855506	0.528225	1.337599	1.007599	2.833970
Pressure and Elevation series → Precipitation series	0.921488	0.822895	0.397419	0.397419	2.318683
Temperature series → Precipitation series	0.865830	0.857891	0.033855	0.033855	25.574971

### 3.3 GIS-based modeling results

**Fig. 13:**
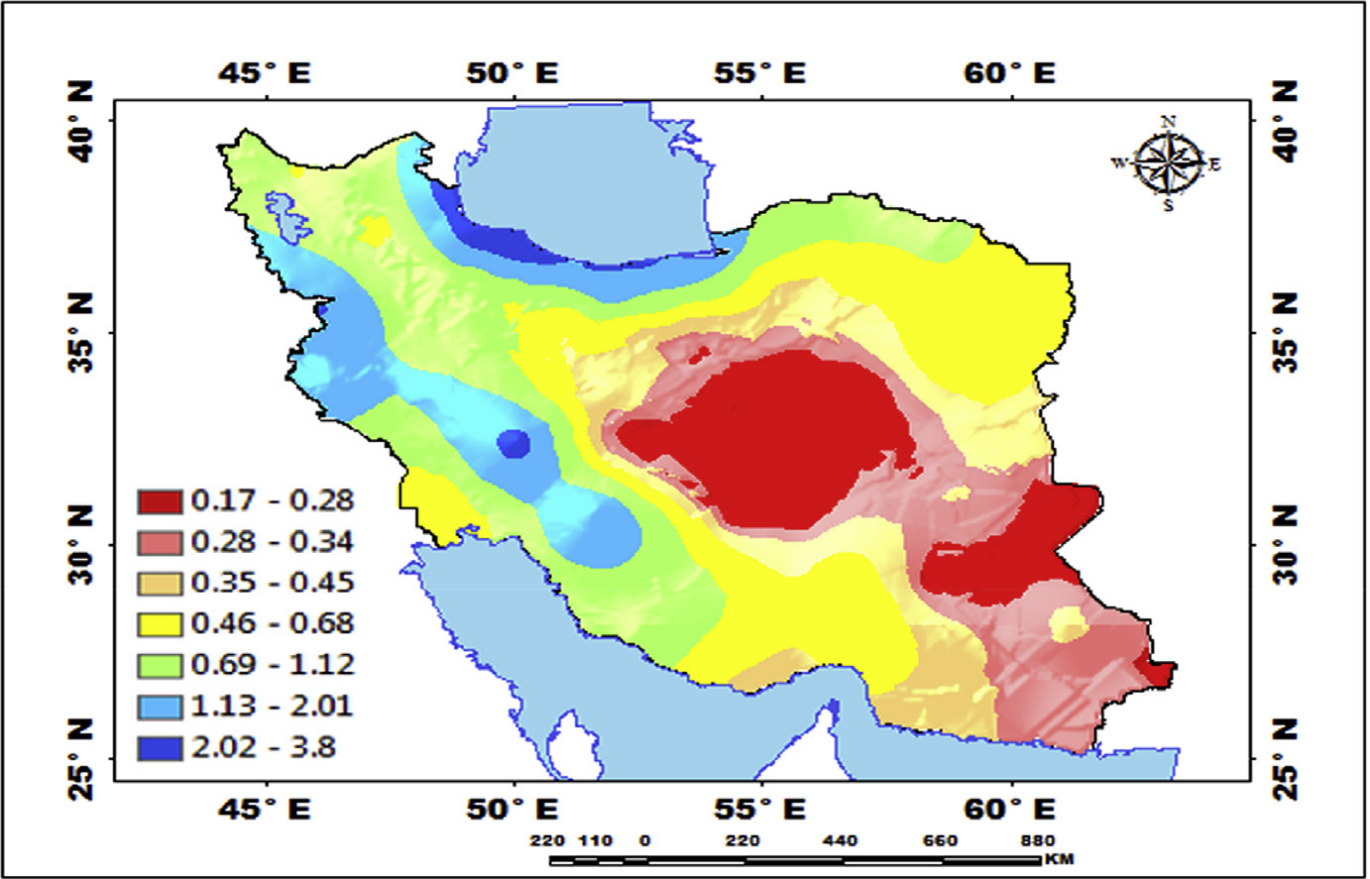
Distribution of the daily precipitation series in Iran.

**Fig. 14:**
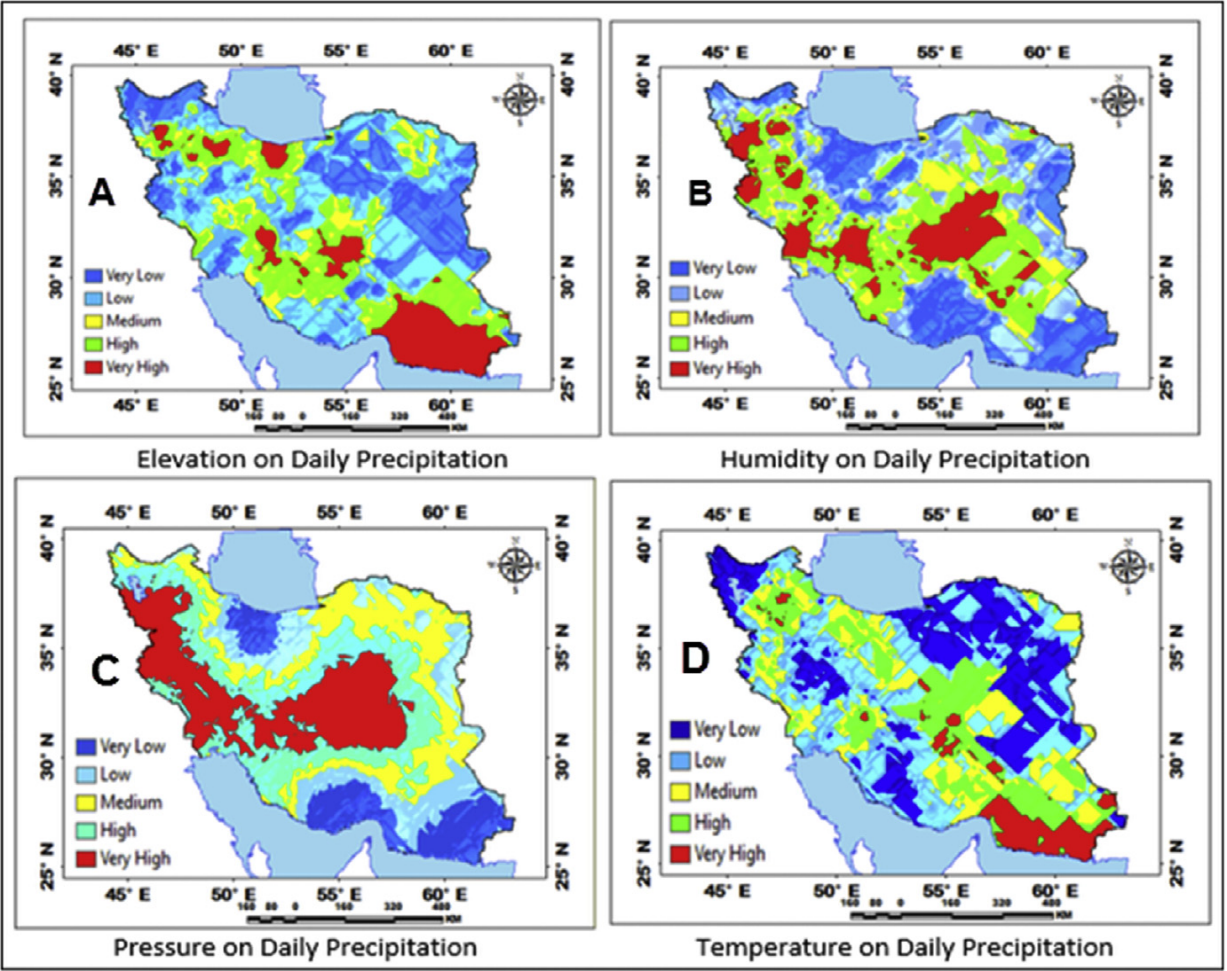
Spatial effectiveness variability of the climatic series on daily precipitation using PLS-algorithms-based final modeling in Iran.

**Fig. 15:**
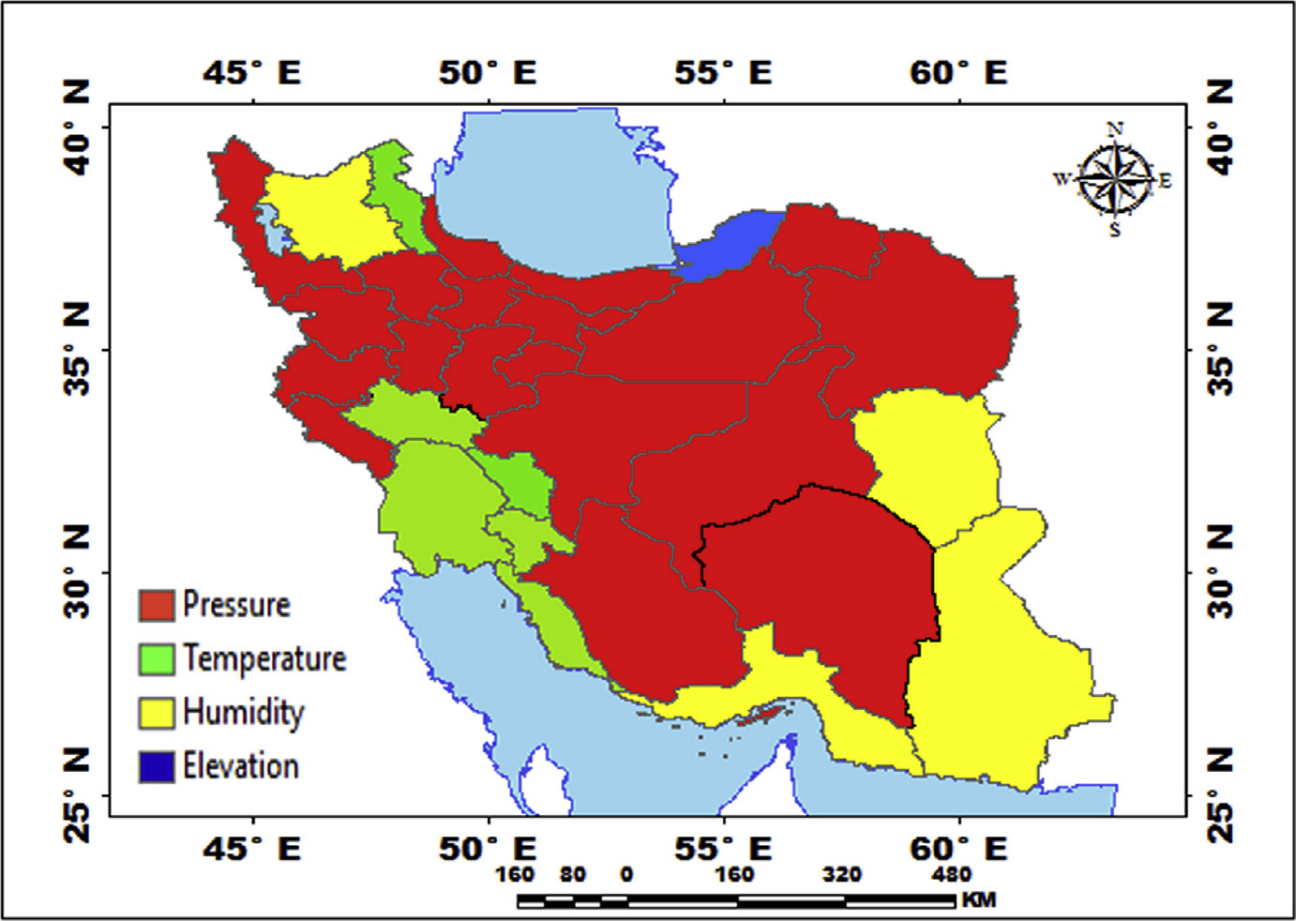
Spatial effectiveness frequency of the climatic series on daily precipitation using Comprehensive-based modeling in Iran.

In this stage, we consider the results from the used CPSRM-based and PLS-based modeling techniques (reflective, formative, and hybrid patterns) to map the findings extracted from the CPSRM-based and PLS-based modeling. We describe the spatial patterns of the considered models and results as well as exploratory findings. In this stage, we focus on climatic effect patterns spatially such as the impacts of temperature indicators on rainfall, the impacts of humidity indicators on rainfall, and the impacts of elevation and pressures indicators on rainfall in Iran. We use GIS-based spatial-interpolation techniques such as Kriging methods to analyze the effect results of the CB-CPSRM-based and PLS-based models. Considering the total effects based on CPSRM and PLS-based modeling in climate effect in Iran, the study focuses on application of the FGT-SGT models to assess the effect of the final model as shown in Fig. 14. Fig. 14 shows the spatial distribution of the effect of climatic series on daily precipitation using hybrid (final) model in Iran from 1975 to 2014. Fig. 14 shows five effect levels on application of the FGT-SGT models for the final model in the GIS-based modeling. The spatial variability indicates the range of effect on climatic series on daily precipitation occurred in Iran, varying from very low to very high. The range of effect in Iran is different based on the elevation factor, especially in the Zagros and Alborz mountains regions. Some CPSRM accept that elevation impact on precipitation over the Caspian Sea is less important than those of the Zagros and Alborz mountains regions due to some strong synoptic systems (effect by humid westerly winds). In addition, as Fig. 14A noticeably shows, the rate of effect of the elevation factor on daily precipitation in southeast and central parts of the country is greater than that of the northeast parts. The Zagros eastern slopes, Azerbaijan mountains, and Makran mountains play an important role in effect of the elevation factor on daily precipitation in central, northwest, and southeast parts of the country. However, in effect of the elevation factor on daily precipitation, there exists no individual pattern in Iran. The indicator point of the maximum effect of the elevation factor on daily precipitation over the coastal regions of the Oman Sea is situated in Sistan and Baluchistan with a less important number of rainy days. In this region toward the south, the value of rainfall distribution has increased (Fig. 13). The Oman Sea coasts is located along a direction affected by humid Seasonal winds (monsoon low pressure system), and then the lowland area is controlled by the Makran Mountains (Fig. 14A). Thus, there is decreased irregular effect of the elevation factor on daily precipitation northward from the Oman Sea coast and northeastward in Iran. However, in central and northwest regions, the decreased irregular effect is modified due to the Alborz and central mountains. Secondary components of the effect of the humidity factor on daily precipitation, in the western and central regions, play a crucial role in effect of the humidity factor on daily precipitation (Fig. 14B). Fig. 14B shows the effect spatial variability of the humidity variables on daily precipitation in Iran during the period 1975–2014. Compared to the elevation factor, effect of the humidity variables shows a northern and western shift of effect during the period 1975–2014. In Fig. 14B, in two realms, the maximum effect of the humidity variables corresponds with central and western regions. Such effect spatial variability patterns can appear in this realm as a result of a supported and shifted humid seasonal winds system (monsoon winds system) and the humid western winds (prevailing westerly's system) into a hydrodynamic process (Fig. 14B). Furthermore, Fig. 14B shows that the area of the maximum effect of the humidity variables in western slopes of the Zagros Mountains is greater than that of the central regions. The western slopes of the Zagros Mountains play an important role in absorbing rainfall and humidity by western winds or prevailing westerly's system, a system that can be the influx of Mediterranean cyclones. A realm linked to the distribution of maximum precipitation is situated in the western zones of Iran. In addition, Fig. 14B shows the area of minimum effect of the humidity variables in northern slopes of the Alborz Mountains, southern coasts, and northeast regions. The zonal conditions and prevailing high-pressure system (Siberian anticyclone) play an important role in decreasing effect of the humidity variables on daily precipitation, a pattern that can be due to lack of flow humidity resources. Fig. 14C shows effect spatial variability of the pressure variables (minimum pressures series) on daily precipitation in Iran during the 1975–2014 period (Fig. 11). The effect spatial variability of the pressure variables shows a higher effect zone in the Zagros Mountains and central regions during the period 1975–2014. The different effectiveness of climatic series on precipitation distribution in the predicted series has altered spatiotemporally such that pressure variables has been monitored in the west and central parts of the country and temperature series distribution belongs to the Oman sea coastline, southeast regions of Iran (Fig. 14). The results of the causal modeling investigation revealed that effectiveness of climatic series on precipitation indicated a strong irregularity in climatic variables for precipitation variability in Iran. In addition, The focus of the effect spatial variability of the pressure variables on daily precipitation is observed in the impact zone of the prevailing westerly's system and low pressure of the Zagros mountains, with its spatial variability rate being observably decreased as one pattern toward other parts (Fig. 14C). As Fig. 14C shows, in one region, the maximum effect spatial variability of the pressure variables on daily precipitation corresponds with intense precipitation. Fig. 14D shows effect spatial variability of the temperature variables on daily precipitation in Iran. Fig. 14D shows that the effect rate of effect is mainly examined over the coasts of the Oman Sea. Additionally, Fig. 14D shows that the second focus of the effect spatial variability of the temperature on daily precipitation in Iran during 1975–2014 is mainly situated in Azerbaijan regions, especially in East Azerbaijan Province. This effect pattern can be controlled by mechanisms of atmospheric circulation system and local conditions is based on the main dynamics effecting in Azerbaijan regions and coasts of the Oman Sea (Modarres and Sarhadi, 2011; Ghasemi and Khalili, 2006; Raziei et al., 2012; Najafi et al., 2017; Liu et al., 2017). However, the factors effect on daily precipitation has considerable diversity in Iran (Fig. 14). In fact, the diversity of factors effect on daily precipitation is controlling the precipitation distribution in Iran. Assessment of the diversity of factors effect on daily precipitation in the period 1975–2014 shows decreased effect of internal factors than the external factors in Iran. Fig. 15 shows the dominant frequency of the maximum effect in the provinces of Iran. Furthermore, as Fig. 15 shows, the number of stations effect is different for pressure variables (stations 100 or 58.8%), humidity variables (stations 37 or 21.8%), temperature variables (stations 25 or 14.7%), and elevation variable (stations 8 or 4.7%) to extract the estimated values of climatic series in the Comprehensive -based modeling based on the spatial variability produced in its GIS-based modeling. In northern and central regions, the pressure variable effect range on daily precipitation has developed in nineteen provinces. Furthermore, in southern regions, Hormozghan, Sistan und Baluchestan, and South Khorasan provinces of Iran, the rate of humidity effect on daily precipitation has increased. Additionally, the rate of temperature effect on daily precipitation in western regions, Khuzestan and Chaharmahal and Bakhtiari, Kohgihluyeh and Boyer-Ahmad, Lorestan, and Bushehr provinces, and the rate of elevation effect on daily precipitation for Gholston Province have increased. The dominant frequency of the maximum effect of climatic series in these provinces for pressure (58.8%) and humidity (21.8%) on daily precipitation are predicted (80.6%) for pressure and humidity variables. As Fig. 15 shows, the regions with maximum effect on daily precipitation can be considered mainly as factors of formation of precipitation in Iran. The results of effectiveness analysis showed that precipitation distribution and its variability have been influenced by pressure variables (Climatic systems come from the Siberia, Europe, and North Atlantic Ocean) (Fig. 15).

## 4 Conclusions

The results indicated that during the period of 1975–2014, 80.6% of the climatic variables effectiveness had belongs to the pressure and humidity variables distributions, respectively. The results of zoning of the climatic variables effectiveness spatiotemporally revealed that the precipitation variability follows the different patterns in Iran. The patterns and effect frequency of climatic series on daily precipitation in the period 1975–2014 examined in this CPSRM indicate that four effect regions in Iran can be detected for the rate of these effect factors:

1) In northern and central regions, the effect of pressure series on daily precipitation is frequently focused.
2) In the south and southeast along the Oman Sea coasts and Sistan und Belutschistan, and South Khorasan provinces of Iran, effect of humidity series on daily precipitation is increased.
3) In the west and southwest along Khuzestan and Chaharmahal and Bakhtiari, Kohgihluyeh and Boyer-Ahmad, Lorestan, Bushehr, and Ardebil provinces of Iran, effect of temperature series on daily precipitation is increased.
4) In the north and southeast along the Caspian Sea, effect of elevation series on daily precipitation is increased.
5) In the causal modeling, a typical application for effect formative model is supposed in Iran, but the variability in precipitation is more changeable, with various predicting to the climatic causal modeling.

Effect patterns appeared in the dominant frequency of climatic series on daily precipitation are summarized in zonal conditions, and the prevailing high-pressure system plays an important role in the patterns change of effect of the climatic variables on daily precipitation. The results of analyzing the first-generation techniques (FGT), second-generation techniques (SGT), third-generation techniques (TGT), and causal hybrid techniques (CHT) indicate that, although the climatic temporal-spatial effect variability (SEV) has predicted spatial and temporal changes in the precipitation patterns, it has mainly focused on effect of various factors in Iran. The spatiotemporal effect variability (SEV) of climatic factors is not similar to that of rainfall distribution patterns in Iran. Effect spatial variability in daily precipitation shows considerable diversity and composite patterns in recent years. Therefore, according to the spatiotemporal effect variability, analysis of effect and causality (PEC) requires an analytical model to predict the spatiotemporal changes in the precipitation patterns. However, the CHT can help us to detect climatic changes by SEV in Iran and other regions.

## Declarations

### Author contribution statement

Majid Javari: Conceived and designed the experiments; Performed the experiments; Analyzed and interpreted the data; Contributed reagents, materials, analysis tools or data; Wrote the paper.

### Funding statement

This work was supported by research management in Payame Noor University (1396).

### Competing interest statement

The author declares no conflict of interest.

### Additional information

No additional information is available for this paper.
